# Multilocus Phylogeography and Species Delimitation in the Cumberland Plateau Salamander, *Plethodon kentucki*: Incongruence among Data Sets and Methods

**DOI:** 10.1371/journal.pone.0150022

**Published:** 2016-03-14

**Authors:** Shawn R. Kuchta, Ashley D. Brown, Paul E. Converse, Richard Highton

**Affiliations:** 1 Department of Biological Sciences, Ohio Center for Ecology and Evolutionary Studies, Ohio University, Athens, Ohio, United States of America; 2 Department of Biology, University of Maryland, College Park, Maryland, United States of America; University of Colorado, UNITED STATES

## Abstract

Species are a fundamental unit of biodiversity, yet can be challenging to delimit objectively. This is particularly true of species complexes characterized by high levels of population genetic structure, hybridization between genetic groups, isolation by distance, and limited phenotypic variation. Previous work on the Cumberland Plateau Salamander, *Plethodon kentucki*, suggested that it might constitute a species complex despite occupying a relatively small geographic range. To examine this hypothesis, we sampled 135 individuals from 43 populations, and used four mitochondrial loci and five nuclear loci (5693 base pairs) to quantify phylogeographic structure and probe for cryptic species diversity. Rates of evolution for each locus were inferred using the multidistribute package, and time calibrated gene trees and species trees were inferred using BEAST 2 and *BEAST 2, respectively. Because the parameter space relevant for species delimitation is large and complex, and all methods make simplifying assumptions that may lead them to fail, we conducted an array of analyses. Our assumption was that strongly supported species would be congruent across methods. Putative species were first delimited using a Bayesian implementation of the GMYC model (bGMYC), Geneland, and Brownie. We then validated these species using the genealogical sorting index and BPP. We found substantial phylogeographic diversity using mtDNA, including four divergent clades and an inferred common ancestor at 14.9 myr (95% HPD: 10.8–19.7 myr). By contrast, this diversity was not corroborated by nuclear sequence data, which exhibited low levels of variation and weak phylogeographic structure. Species trees estimated a far younger root than did the mtDNA data, closer to 1.0 myr old. Mutually exclusive putative species were identified by the different approaches. Possible causes of data set discordance, and the problem of species delimitation in complexes with high levels of population structure and introgressive hybridization, are discussed.

## Introduction

As species are fundamental units in ecology, biodiversity, conservation, and evolutionary biology, accurate species delimitation is of critical importance. Nonetheless, the diagnosis of species has a contentious history, with many biologists advocating alternative species concepts and conflicting taxonomies [1–5]. Work on species delimitation has historically focused on morphology and patterns of reproductive isolation [6–8], but molecular techniques are increasingly used to clarify species boundaries [9–13]. Using molecular markers, many morphologically cryptic taxa have been identified, and deep divergences among allopatric components quantified [14–17]. While species delimitation remains a challenging, philosophically rich topic [3,5], increasing numbers of biologists—especially systematists—are conceptualizing species as segments of independently evolving metapopulation-level evolutionary lineages, a perspective known as the general lineage species concept [4,18,19]. In particular, this perspective has been widely applied to molecular systematic investigations of complexes characterized by allopatry, parapatry, and morphological stasis [3,20–22]. In conjunction with advances in developing a unified concept of species [23], multispecies coalescent models, which lie at the interface of modern population genetic and phylogenetic methods, are revamping the science of species delimitation [24–26]. The development of these models was spurred by the observation that genealogies estimated from different genes can be discordant simply because the coalescence of genealogical lineages is a stochastic process. Unlike concatenated phylogenetic analyses, which assume a single tree underlies all loci, the multispecies coalescent accounts for gene tree conflict by modeling coalescent stochasticity [24,27]. Thus, evolutionary lineages can be diagnosed, and a reliable estimate of a species tree made, in the absence of monophyly, or when the information content of many loci is weak [28,29]. On the other hand, serious complications such as introgressive hybridization, high levels of population structure, isolation by distance, and phylogenetic estimation error present analytical challenges for genetic data, and if not accounted for can mislead inferences [25,30,31].

Species limits are notoriously difficult to identify in plethodontid salamanders, which can exhibit a high degree of phenotypic and ecological conservatism [32–35], yet commonly harbor extraordinary levels of genetic variation, including isolation by distance and deep population structure [9,36–41]. Objectively defining and delimiting plethodontid species is therefore a challenging task [3,42–44]. Nonetheless, largely as a result of studies using allozymes, the number of species in the genus *Plethodon* has increased from 16 in 1962 [45] to 55 today (AmphibiaWeb: http://amphibiaweb.org), including numerous cryptic and allopatric species [9,37,46]. Species complexes in *Plethodon* are thus eminent examples of a non-adaptive radiation, whereby an ancestral source taxon disintegrates into a complex of isolated lineages [46–49].

**Fig 1:**
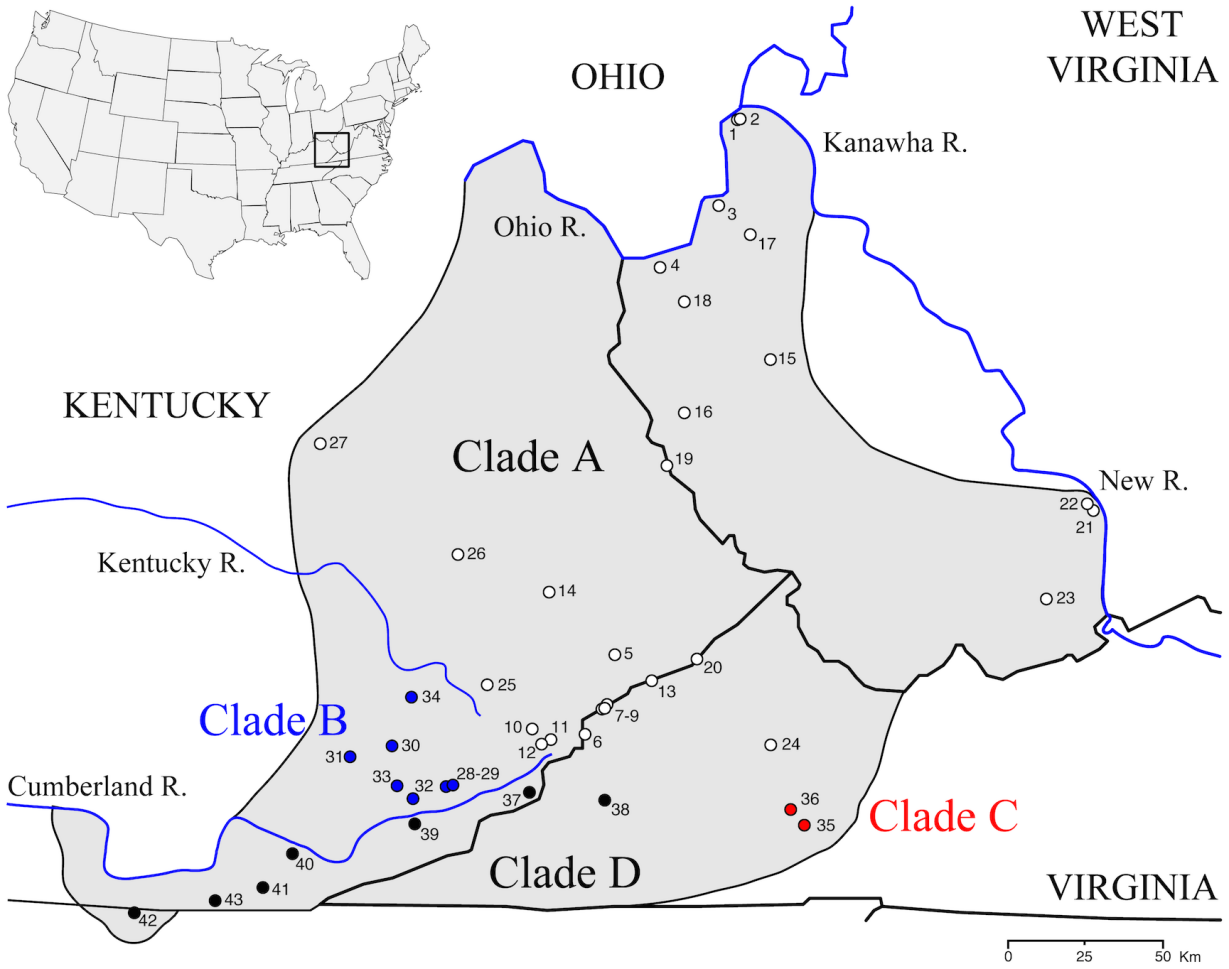
Map of the range of *Plethodon kentucki*. Sample localities are numbered, and match Fig 3 and S1 Appendix. Symbols identify the mtDNA clade of individuals in that population (Fig 3).

The Cumberland Plateau Salamander, *P*. *kentucki*, is an example of a species of *Plethodon* that harbors a high degree of genetic structure across its range. The species was originally described by Mittleman in 1951 [50]. However, it was not found to be morphologically distinct [51] and the name was long regarded as a junior synonym of *P*. *glutinosus*. In 1983, the species was rediscovered by Highton and MacGregor [52] while surveying patterns of genetic (allozyme) variation in the *Plethodon glutinosus* complex [9]. Geographic surveys showed that *P*. *glutinosus* and *P*. *kentucki* co-occur over most of the range of *P*. *kentucki*, though the range of *P*. *kentucki* is relatively restricted, including western Kentucky, southwestern West Virginia (south of the New and Kanawha Rivers), western Virginia, and a small section of northern Tennessee (Fig 1). Both species possess a black ground color overlain with white spots. Living specimens can be identified by subtle differences in *P*. *kentucki*, such as a lighter chin, a smaller number of dorsal spots, and a distinctive shape of the mental gland. However, the differences are quantitative, and within populations ranges of phenotypic variation between the two species can overlap.

Despite the restricted range of *P*. *kentucki*, Highton and MacGregor [52] documented substantial amounts of genetic diversity. An electrophoretic analysis of 22 presumptive genetic loci showed that Nei’s [53] genetic distances were as high as 0.43 among geographically widespread samples. This is interesting because Highton [43] has argued that D_N_ ≥ 0.15 commonly separates distinct species, while Wake and Schneider [44], counter to Highton, cite *P*. *kentucki* as an example of a single species with high levels of genetic differentiation. Here, we revisit patterns of genetic variation within *P*. *kentucki* using multilocus sequence data.

Species formation is a time-extended process, potentially including incomplete lineage sorting, introgressive hybridization, the anastomosis of formerly isolated lineages, and the development of complex patterns of population structure among incompletely separated lineages [3]. Thus, the parameter space relevant for species delimitation is large and complex. By contrast, all methods of species delimitation make a number of simplifying assumptions that may lead them to fail when faced with real world data sets [31,54]. In this paper we apply an array of approaches to species delimitation within *P*. *kentucki*, with the assumption that strongly supported species, at least, will we be recovered by alternative methods [11,31]. In this respect the approach is conservative. Incongruence among methods can be due to differences in their power to detect cryptic genetic lineages, or can be artifacts resulting from the violations of assumptions. We used three approaches to delimiting putative species. First, for mtDNA sequence data we employed a Bayesian implementation of the GMYC model [55]. Next, we used Brownie and Geneland [56–59] to delimit species using our nuclear data. Putative species were then validated using the genealogical sorting index, or *gsi* [60], and the program Bayesian Phylogenetics and Phylogeography (BPP). This latter approach uses the multispecies coalescent to analyze DNA sequence data, and can accommodate incomplete lineage sorting and uncertainty in the topology of the species tree [24,61,62]. We found that different methods were wildly inconsistent and advanced mutually exclusive taxonomies. We propose that this is a consequence of introgressive hybridization, isolation by distance, and high levels of population structure, and that *P*. *kentucki* may represent a particularly challenging real world scenario for modern species delimitation methods.

## Materials and Methods

### Natural history

The Cumberland Plateau Salamander, *Plethodon kentucki*, is a Woodland salamander in the family Plethodontidae. Unlike many amphibians, Woodland salamanders have no aquatic larval stage, do not migrate, and are completely terrestrial. Territoriality and home range size have not been studied in *P*. *kentucki*, but in *Plethodon* in general home ranges are small, on the order of a few square meters [63–65]. Small home ranges, territoriality, and limited mobility promote the accumulation of genetic differences among populations, including high levels of phylogeographic structure [40,41,66,67]. The systematic history of *P*. *kentucki* is reviewed in detail by Highton and MacGregor [52].

### Sampling and laboratory techniques

Blood samples and tail tips were collected from 135 individuals from 43 populations of *P*. *kentucki* (Fig 1). Blood samples, designated with RH numbers in S1 Appendix, were collected from euthanized specimens in the early 1980s, prior to IACUC [52]. Animals were sacrificed by immersion in a solution of chlorotone before blood was collected. In addition, more recently about 3 mm of tail tip were collected from live specimens under Ohio University IACUC 12-L-050; these are designated with SRK numbers in S1 Appendix. Permits were obtained for our field efforts from the Virginia Department of Game and Inland Fisheries (015603, 048037), the West Virginia Division of Natural Resources (2013.115), and the Kentucky Department of Fish and Wildlife (SC1311198). Sampling was oriented toward geographic coverage and describing the limits of haplotype lineages. Total genomic DNA was extracted using Qiagen DNeasy Blood and Tissue Kits (Qiagen Corp., Valencia, CA). A total of 5693 base pairs (bp) of DNA were sequenced. Mitochondrial DNA sequence data were collected from most of the cytochrome *b* gene (Cyt-*b*; 1105 bp), the complete NADH dehydrogenase 2 gene (ND2; 1041 bp), the complete tRNA^*trp*^ locus (66 bp), and a portion of tRNA^*ala*^ (33 bp). ND2 was sequenced in two overlapping parts. MtDNA sequence data were collected from all but one individual (RH62903, S1 Appendix), but we have mtDNA data from two other individuals from that population (population 22). DNA sequence data were collected for five nuclear loci: the nuclear exon recombination activating gene 1 (RAG-1; 1152 bp), and the nuclear introns interleukin enhancer binding factor 3 (ILF3; 251bp), myosin light chain 2 mRNA (MLC2A; 416 bp), glyceraldehyde-3-phosphate dehydrogenase (GAPD; 686 bp), and *β*-fibrinogen intron 7 (BFI; 943 bp). BFI was amplified using a two-step protocol in which an initial long segment was first amplified from genomic DNA, then the product of this reaction was used as template in a subsequent PCR reaction, as described by [68]. This is the first study to use this gene in a plethodontid salamander. To our data set we added previously published sequence data from two individuals: ND2 for one individual from [69], and sequence data for one individual for Cyt-*b*, RAG-1, ILF3, MLC2A, and GAPD from [49,70]. Primers for all loci are provided in Table 1. Nuclear sequence data were collected from a subset of individuals, which varied by locus (Table 2). Details regarding the sampling of populations and loci, geographic coordinates, and GenBank accession numbers are presented in S1 Appendix.

**Table 1:** Primer sequences for all loci used in the study.

Locus	Primer Name	Nucleotide Sequence (5'– 3')	Reference
Cyt-*b*	Pglut-F1b	GGTCTGAAAAACCAATGTTGTATTC	[71]
	PThr-R2b	GCCCCCAATTTTGGYTTACAAG	[71]
ND2 +	ND2-L4437-F2	AAGCTTTCGGGCCCATACC	[72]
tRNA^ala^ +	ND2-RNEW1DEG	ATCCYAGGTGRGCGATGGAGG	This study
tRNA^trp^	ND2-L5195-F3	TGACAAAANCTNGCCCC	[69]
	ND2-Ra	GTCTTGCAAGTTCGAGTCAGA	[73]
	ND2-5200	CCTTGCCCTCTCATCCAAATCAGC	This study
	KND2-R2	AAAGTGTTTGAGTTGCATTCA	[74]
RAG-1	Rag-1b-F	CTGTCTGGTCTGGTGCAGTCG	This study
	Rag-1a-R	ATTCCCTTCACTCGCCCAAGC	This study
BFI	BFI-EF1	GGAGANAACAGNACNATGACAATNCAC	[68]
	BFI-ER1	ATCTNCCATTAGGNTTGGCTGCATGGC	[68]
GAPD	GAPD-F	ACCTTTAATGCGGGTGCTGGCATTGC	[70]
	GAPD-R	CATCAAGTCCACAACACGGTTGCTGTA	[70]
ILF3	ILF3-F	GATTTCAATCCATTTGCTCTTGC	[70]
	ILF3-R	AGGATAAGCCCACCGTTACACTATT	[70]
MLC2A	McL2a-F5	TCCAATGTCTTTGCCATGTTCG	This study
	MCL2a-R2	AGTCATCCTTGTCTTTGGCTCC	This study

**Table 2:** Sampling, genetic diversity, and models of evolution. For the models of evolution, "All data" refers to the inclusion of every DNA sequence, whereas the “Complete data set" refers to the data set with no missing data (68 individuals). Numbers in parentheses refer to codon positions.

Locus	Length^1^	No. Indiv.^2^	No. pops	P	Unique haplo-types	*h*	π	κ	Model of evolution: All data	Model of evolution: Complete data set
mtDNA	5693	––	––	––	––	––	––	––	ND2(1), Cyt b(1), tRNAs: GTR + I + Γ; ND2(2), Cyt b(2): TN93 + I; Cytb(3): TN93; ND2(3): GTR + Γ	ND2(1), Cyt b(1), tRNAs: GTR + I; Cyt b(2): HKY + I; Cyt b(3): TN93; ND2(2): TN93 + I; ND2(3): GTR + Γ
Cyt b	1105(973)	133(129)	43(43)	154	39	0.96	0.037	35.7	(1)GTR + I, (2)HKY + I, (3)TN93	GTR + I
ND2	1041(977)	85(77)	43(43)	169	43	0.98	0.043	41.6	(1)GTR + I, (2)TN93 + I, (3)GTR + Γ	TN93 + Γ + I
tRNA^trp^	66(64)	79(76)	42(42)	4	5	0.35	0.007	0.5	HKY	HKY
tRNA^ala^	33(33)	73(72)	42(42)	10	5	0.59	0.101	3.3	GTR + I	GTR + I
RAG-1	1152(1145)	74	42	30	30	0.67	0.001	1.6	HKY + I	HKY + I
BFI	943(898)	75	42	46	37	0.64	0.001	1.2	HKY + I	HKY + I
GAPD	686(383)	77(74)	42	20	18	0.86	0.008	3.1	HKY + Γ	HKY + Γ
MLC2A	416	80(79)	43(43)	23	23	0.75	0.005	1.6	JC + Γ	JC + Γ
ILF3	251	73(69)	42(41)	10	10	0.66	0.005	1.1	JC	JC

Diversity indices: P, the number of polymorphic sites; Unique haplotypes: the number of unique haplotypes in the sample; haplotype diversity, *h*, the probability that two randomly selected haplotypes are different from each other; nucleotide diversity, π, the average number of nucleotide differences per site between two sequences; sequence diversity, κ, the average number of nucleotide differences between paired sequences [78].

^1.^ Sequence length. Numbers in parentheses are the length of the sequence used to calculate diversity indices after omitted all sites with missing data.

^2.^ Number of individuals. Numbers in parentheses are after excluding short sequences, which were not used to calculate diversity indices because they exclude sites with missing data.

Most samples were sequenced in both the forward and reverse direction. The exception is ND2, which was only sequenced in the forward direction. Electropherograms for all sequences were viewed using the program Geneious v6.1 (Biomatters, Ltd., San Francisco, CA), and ambiguous base calls were manually corrected. The phase of heterozygous genotypes was estimated using PHASE v2.1.1 [75]. We ran PHASE for 1000 iterations, with a thinning interval of two steps and a burn-in of 100 iterations. PCR products exhibiting length heterogeneity due to the presence of indels were phased using Champuru v.1.0 [76,77]. In a few instances, sequences that could not be resolved using Champuru were cloned using the Invitrogen TOPO-TA Cloning Kit (Invitrogen, Carlsbad, CA). Four separate colonies were sequenced from each clone, and in all cases the heterozygotes were resolved. We tested for intragenic recombination using the difference in sum-of-squares (DSS) test implemented in TOPALi [78], including a 10 base pair increment, a window size of 100, and 500 parametric bootstraps. Recombination was not detected at any locus.

We used genetic diversity indices to compare patterns of genetic differentiation among mtDNA clades. Diversity indices included polymorphism (P), the number of segregating sites (S), haplotype diversity (h), sequence diversity (κ), and nucleotide diversity (π) [79]. Calculations were carried out in DNAsp5.10.1 [80].

### Rates of Evolution

To obtain a time calibrated phylogeny it is necessary to either date nodes or provide an estimate of the rate of evolution. Currently it is not possible to date any of the nodes within *P*. *kentucki*, as there are no fossils or dated biogeographic events [81]. Thus, we estimated rates of evolution using Bayesian relaxed-clock dating with PAML 4.1 [82] and the set of programs in the Multidistribute package [83–85]. For each locus, *baseml* was used to estimate parameters under the F84+Γ model of nucleotide substitution, and *paml2modelinf* was used to transform output from *baseml* into a format appropriate for downstream analyses. The program *estbranches* was used to obtain maximum likelihood estimates of branch lengths and the variance-covariance matrix of those estimates. Finally, *multidivtime* was used to approximate the posterior distributions of substitution rates for each locus. These programs were run in Unix and through the R package LOGOPUS [86].

We estimated rates of evolution for Cyt-*b*, ND2, tRNA^*trp*^ and tRNA^*trp*^, RAG-1, MLC2A, and GAPD using the phylogeny of Pyron and Wiens [87] trimmed to include only the genus *Plethodon*. The GenBank accession numbers and phylogenies used to estimate rates of evolution are provided in S2 Appendix. Species in the subgenus *Hightonia* (the clade of *Plethodon* restricted to the western US) served as outgroup taxa [88]. The species *P*. *glutinosus*, *P*. *shermani*, and *P*. *aureolus* were excluded because some analyses suggested they may be paraphyletic [70,89]. Clade age calibrations were taken from Wiens et al. [49], which were derived from three possible crown group ages for the family Plethodontidae of 50, 66, and 85 myr. We ran analyses using all three age estimates (Table 3). Priors for the mean (standard deviation) of the ingroup root age were 18.96 (1.17), 25.12 (1.46), and 32.25 (1.97) myr for crown group ages of 50, 66, and 85 myr, respectively. Time calibrated nodes included the *cinereus* group, *glutinosus* group, *welleri-wehrlei* group, and *ouachitae* group (see [89] for a discussion of group memberships), with upper and lower bounds set at two standard deviations from the mean. Two loci were analyzed separately. For ILF3, we designated the *cinereus* group as the outgroup [70,87,89] due to a lack of sequence data from the subgenus *Hightonia*. Ingroup root ages were estimated at 15, 20, and 25 myr (SD = 3 myr) for the crown group ages of 50, 66, and 85 myr, respectively (estimated from Figure 5 and Table 3 in [49]), and dated nodes included the *glutinosus* group, the *welleri-wehrlei* group, and the *ouachitae* group. For BFI, few sequences were available outside of *P*. *kentucki*. Thus, we estimated the rate of evolution using a three-taxon statement, with P. *wehrlei* as the outgroup and *P*. *glutinous + P*. *kentucki* as the ingroup. The ingroup age was set at 9.79 (SD = 0.3) myr [49]. Because this estimate of molecular evolution was derived from limited sampling, we also estimated the rate of evolution for BFI using a phylogeny and sequence data from the family Salamandridae, as described in S2 Appendix.

**Table 3:** Estimates of median rates of evolution. Three estimates of the age of the crown group of plethodontids were used [71]. 95% confidence intervals are in parentheses. For clarity, all values are multiplied by 100, and thus are the estimated percentage change per million years.

	50 Myr	66 Myr	85 Myr
**Cyt-*b***	0.659	0.623	0.496
	(0.444, 0.912)	(0.424, 0.868)	(0.337, 0.696)
**ND2**	0.889	0.844	0.665
	(0.629, 1.167)	(0.611, 1.088)	(0.483, 0.862)
**tRNAs**	0.149	0.127	0.099
	(0.016, 0.358)	(0.010, 0.324)	(0.007, 0.270)
**MLC2A**	0.083	0.074	0.061
	(0.012, 0.213)	(0.009, 0.195)	(0.007, 0.157)
**GAPD**	0.077	0.072	0.058
	(0.023, 0.164)	(0.023, 0.153)	(0.017, 0.126)
**RAG-1**	0.029	0.026	0.023
	(0.011, 0.051)	(0.011, 0.046)	(0.009, 0.037)
**ILF3**	0.102	0.074	0.043
	(0.022, 0.225)	(0.012, 0.173)	(0.005, 0.117)
**BFI**	——	0.036	——
		(0.006, 0.081)	

### Phylogenetic Analyses

Gene trees were inferred using Bayesian phylogenetic analysis. We first analyzed all the data for each gene separately, without removing identical haplotypes [90], and including both alleles for nuclear loci. Because some methods do not accommodate missing data, and to facilitate comparison among loci, we also assembled a “complete data set” in which every OTU included data from every locus. We allowed missing data in either Cyt-*b* or ND2 (but not both) because these loci constitute a non-recombining unit. The complete data set included 68 individuals from 42 populations from throughout the range of the *P*. *kentucki*. For most populations, two individuals were included; only populations 1 and 18 are not represented in the complete data set (S1 Appendix).

For nuclear loci, models of evolution were assessed using jModeltest 2.1.5 [91], with the best model selected using AICc (Table 2). Models of evolution and the partitioning scheme for the concatenated mtDNA data were determined using PartitionFinder v1.1.1 [92] (Table 2). For all gene trees, we used a constant population size coalescent tree prior and a strict clock model. Clock models included a lognormal distribution with means and 95% confidence intervals that matched our multidivtime analyses (Table 3). In our preliminary analyses, the default priors (gamma) for *rate*.*CG* and *rate*.*GT*, though themselves well sampled (Effective Sample Sizes [ESS] > 200), resulted in very low ESS values for the prior and posterior distributions, even with long MCMC runs. The use of a lognormal prior on *rate*.*CG* and *rate*.*GT* increased the ESS values for the prior and posterior distributions to >6500. SRK conducted >200 separate runs in BEAST 2 before figuring this out.

Tree models were linked across partitions for mtDNA. For gene tree analyses, the length of the Markov chain Monte Carlo (MCMC) run was set to 50 million generations with parameters sampled every 5000 generations and a burn-in of 25%. All ESS values in all runs were >200. The Maximum Clade Credibility (MCC) tree was chosen using TreeAnnotator 2.1.2 [93].

To account for incomplete lineage sorting and provide input trees for downstream analyses, we performed species tree analyses using the multispecies coalescent model implemented in *BEAST 2 [28,94]. We defined species based on the bGMYC and Geneland results (see below). Analyses in *BEAST were set up as described above, except we used a Yule model for the tree prior and ran analyses for 500 million generations.

Finally, a maximum likelihood (ML) analysis of the concatenated data was conducted using RAxML v.8.1 [95]. We analyzed two data sets, one the nuclear DNA only (3438 bp), and one with nuclear DNA and mtDNA combined (5683 bp). Four individuals of *P*. *glutinosus* were used as the outgroup (S1 Appendix). For both data sets, we conducted 200 heuristic searches to obtain the ML tree, and 1000 rapid bootstrap pseudoreplicates to assess nodal support. The GTRGAMMA model was used, and protein coding loci were partitioned by codon.

### Delimiting putative species

#### bGMYC

We first delimited putative species using a version of the general mixed Yule-coalescent (GMYC) [96]. Given a gene tree, this model infers on a phylogeny the transition from population-level (coalescent) processes to species-level (Yule model) processes. We used a Bayesian extension of this model, called the bGMYC, that accounts for uncertainty in gene trees by sampling over a posterior distribution of sampled trees [55]. The GMYC model is advantageous for single-locus datasets, and when the majority of phylogenetic signal is found in mtDNA, as in our data (see below). bGMYC analyses were run in the eponymous R package ‘bGMYC’ [55]. For the analyses, we randomly subsampled 1000 trees from the posterior distribution of our BEAST 2 analysis of the mtDNA data set. The following run options were used: MCMC = 100,000, burn-in = 50,000, thinning = 200, default scale parameters, default values on the Yule and coalescent rate change priors, and upper and lower bounds on the threshold parameter of 1 and 136, respectively, where 136 was the number of tips in our mtDNA tree. The starting number of species was set to 68, midway between the minimum and maximum number of species.

#### Geneland

To gain insight into population structure within *P*. *kentucki* using our nuclear loci, and for comparison with the bGMYC results, we used Geneland v4.0.5 [56–58]. This spatial clustering program estimates the number of populations by finding the number of groups that maximizes Hardy—Weinberg equilibrium within loci while minimizing linkage disequilibrium between loci. In addition, Geneland accounts for the spatial structure of samples when sampling coordinates are provided. Because missing data can bias the results, we used our complete data set, which was a genotype matrix of 68 diploid individuals (136 alleles) at five nuclear loci. We used the uncorrelated allele frequencies spatial model, as the correlated allele model is best used when differentiation is subtle, and model assumptions, such as no isolation by distance, are met [97]. The number of populations ranged from 1–30, and the MCMC was run for 20 million iterations, with sampling every 1000 steps and the first 20% of steps discarded as burn-in. All runs were replicated 10 times.

#### Brownie

Brownie identifies species limits by maximizing incongruence between gene trees within species, while minimizing incongruence between species. The logic is that within a species gene tree topologies will be random draws from a coalescent process, whereas between species gene trees will often show the same or similar topology. For input, we first randomly sampled one allele from each individual [11,98]. To examine the impact of this random sampling on inference, we analyzed four different samples of alleles. Two of the data sets, which we call data sets 1 and 2, did not include any shared alleles for heterozygous genotypes (e.g. if allele A at a locus was included in one dataset, allele B was used in the other). Data sets 3 and 4 were completely random relative to the other data sets. To make these data sets, we inferred calibrated gene trees in BEAST 2 using all alleles, as described above, and then pruned tips from these trees. We adopted this approach because we assume the accuracy of gene tree inference is increased through the inclusion of all available data. We also conducted analyses on these data sets with mtDNA included, and on the diploid data set without mtDNA. Heuristic searches in Brownie were run using default settings, except that all possible taxon reassignments on leaf splits were explored (Subsample = 1), and the minimum number of samples per species (MinSamp) was set to 2. We conducted 500 independent runs of each data set, and saved the complete set of recovered species trees and species delimitations.

### Validation of putative species

The validity of delimitations inferred using the bGMYC and Geneland was tested using two approaches. First, we tested delimitations against a null hypothesis of no divergence using the genealogical sorting index (*gsi*), which quantifies the degree of exclusive ancestry of labeled groups on a rooted genealogy [60]. Populations in the process of diverging, or that split relatively recently, will usually display mismatches between gene trees and the species tree. However, over time the units are expected to transition from polyphyly to paraphyly to monophyly [99,100]. The time frame of this transition is dependent on the rate of genetic drift, and will vary among neutral loci because lineage sorting is a stochastic process. Relative to nuclear loci, mtDNA is expected to achieve monophyly quicker, on average, because it is haploid and maternally inherited, which results in a lower effective population size and a correspondingly high rate of genetic drift [101,102]. Values for the *gsi* range from 0 to 1, where 0 indicates the absence of exclusive ancestry and 1 indicates monophyly [60]. We calculated *gsi* values for each locus, as well as for an ensemble *gsi* (*egsi*), using the Genealogical Sorting Index web server (http://www.molecularevolution.org/software/phylogenetics/gsi). The null hypothesis of no divergence was evaluated using 10,000 permutations. As uneven sample sizes among groups can shift *P*-values downward for smaller groups, significance was inferred at *P* < 0.01 [60,98,103].

In addition to the *gsi*, the program Bayesian Phylogenetics and Phylogeography (BPP) v3.1 was used to evaluate species delimitations [26]. This program uses the multispecies coalescent to compare species delimitation models while simultaneously inferring a species tree that accounts for incomplete lineage sorting [26,104,105]. Consequently, BPP does not require reciprocal monophyly in gene trees to identify evolutionary lineages. In contrast with earlier versions of BPP, version 3 is not reliant on a fixed guide tree, but rather employs branch swapping with nearest neighbor interchange to alter the guide topology and account for phylogenetic uncertainty. Our analyses included all five nuclear loci, with a single allele sampled at random per individual (data sets 1–4, as described above), with and without mtDNA included. After several exploratory analyses, population size parameters (θ) were assigned the gamma prior G(2, 500), and the divergence time at the root of the species tree (τ) was assigned the gamma prior G(2, 4000); all other divergence time parameters were assigned the Dirichlet prior [104]. We used algorithm 0 with a fine-tune parameter (ε) of 10. Each species delimitation model was assigned equal prior probability. For the MCMC, after a burn-in of 5000 generations, samples were collected every two generations until 20,000 samples were obtained (45,000 generations total). Each analysis was run 2–5 times to confirm consistency among runs.

## Results

### Rates of molecular evolution

As expected, our mtDNA loci showed higher levels of variation than did our nuclear loci (Table 2). For example, sequence diversity (κ) for Cyt-*b* and ND2 was 35.7 and 41.6, respectively, but was 3.1 or less for the five nuclear loci. Mitochondrial tRNAs exhibited lower levels of variation than Cyt-*b* and ND2.

**Fig 2:**
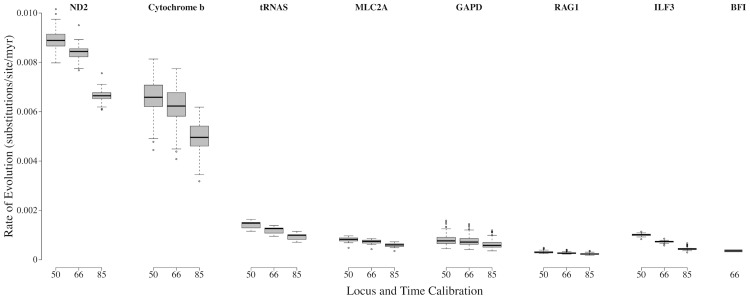
Box plot depicting the estimated rates of evolution of each study locus. Each box plot contains the estimates of evolutionary rate at each node and tip of the tree. The bottom and top of the box delimit the first and third quartiles, respectively, and whiskers extend to a maximum of 1.5 times the interquartile range. Asterisks indicate outlier points.

Median estimated rates of evolution for each gene are shown in Table 3 and Fig 2. Three different estimates are shown, representing three different calibration dates. For the 66 myr calibration, rates of evolution for the mitochondrial loci Cyt-*b* and ND2 were 0.623%/myr and 0.844%/myr, respectively. The tRNAs had a lower rate of 0.127%/myr. Rates of evolution for nuclear loci were roughly an order of magnitude slower than for Cyt-*b* and ND2, and ranged from a low of 0.026%/myr (RAG-1) to a high of 0.074%/myr (MLC2A and ILF3). When BFI was calibrated using *Plethodon*, the estimated rate of evolution was 0.036%/myr, which is an intermediate rate among the nuclear loci. By contrast, when BFI was dated using salamandrids, the estimated rates of evolution were higher than those estimated for our other nuclear loci (details in S2 Appendix). For our analyses, we used the *Plethodon* calibration.

### Phylogenetic inference: gene trees and concatenated analyses

**Fig 3:**
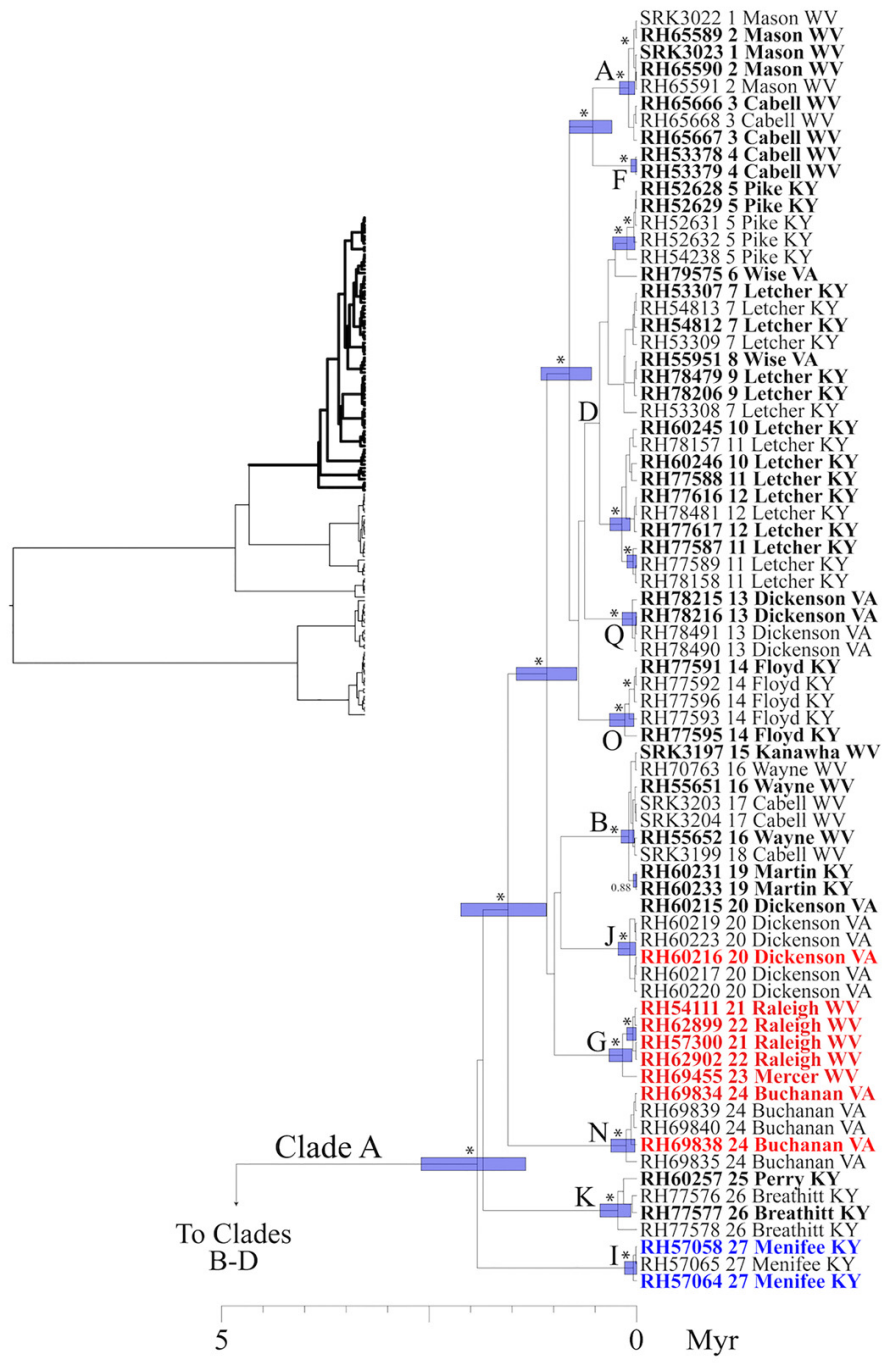
Bayesian maximum clade credibility tree. This phylogeny was inferred using concatenated mtDNA data (Cyt-*b*, ND2, tRNA^trp^, tRNA^ala^). Taxon labels include specimen identification number, the population numbers from Fig 1, and county plus state information. Numbers adjacent to nodes are posterior probabilities (pp), and asterisks identify nodes with pp ≥ 0.95. Bars indicate 95% confidence intervals (CI) for dates of nodes. For visual clarity, many pp values and CI bars were removed near the tips of the tree. Specimens in the "complete" data set, which includes five nuclear loci in addition to mtDNA, are highlighted in bold. The bGMYC analysis delimited either 17 putative species, or two putative species, depending on the probability threshold employed (see text). The 17 putative species are identified using the letters (A-Q) adjacent to nodes; the two putative species are represented by Clade A, and Clades B-C. Finally, three putative species delimited in the Geneland analysis are highlighted using colored text that is either black (species A), red (species B), or blue (species C). Clade A is here illustrated. The entire phylogeny is illustrated in the upper left, with Clade A illustrated using bold lines. See Fig 4 for Clades B-D.

**Fig 4:**
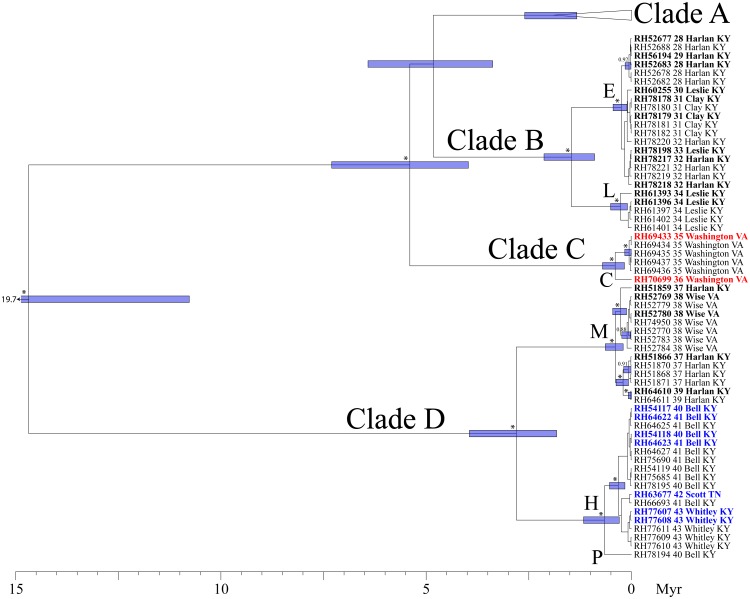
Bayesian maximum clade credibility tree. As with Fig 3, but showing relationships within Clades B-D.

Our analyses of run diagnostics in TRACER suggested that MCMC stationarity was reached in all Bayesian phylogenetic analyses (e.g., all ESS > 200). In our mtDNA gene tree, four primary clades were recovered (Figs 3 and 4). The first split separates Clades A-C from Clade D, and was estimated to have occurred ~14.9 mya (95% HPD: 10.8–19.7 myr). Clade A occupies the broadest distribution of any clade, and is found at the northern and eastern limits of the range, from central Kentucky to eastern Virginia and western West Virginia, west of the New/Kanawha River. This clade harbors the most phylogeographic structure, with a number of subclades of largely unresolved affinity to one another. Clade B (populations 28–34) includes a geographically cohesive group of populations in SE Kentucky, north of the Cumberland River and south of the Kentucky River. Haplotypes from Clade C were found in two populations (35, 36), both of which are restricted to the south. Finally, Clade D occupies a restricted geographic range at the southwest limit of the distribution of *P*. *kentucki*, south of the Cumberland River (Fig 1). It is composed of two subclades, one to the east (populations 37–39), and one to the west (populations 40–43).

Phylogenies for our five nuclear loci, inferred using the complete data set (diploid, no missing loci), are presented in S3 Appendix. In contrast with our mtDNA phylogeny, relationships were poorly resolved. Recovered clades did not circumscribe geographically cohesive groups, and are not similar to any mtDNA clade. For example, in ILF3 four clades had at least moderate support, but none were geographically cohesive or reminiscent of any mtDNA clade. The same is true of RAG-1, BFI, GAPD, and MLC2A: all included supported clades, but these were composed of a mix of alleles from distant geographic localities. Moreover, none of the nuclear loci recovered a clade that was shared with another locus.

The results of maximum likelihood phylogenetic analyses of the concatenated data are presented in S4 Appendix. When the nuclear data alone were analyzed, no statistically supported clades (bs > 70%) were recovered within *P*. *kentucki* (Figure H in S4 Appendix). The three groups recovered in the Geneland analysis of the nuclear data were not covered as reciprocally monophyletic clades, and the clades that were recovered did not form geographically cohesive groups of populations. In addition, one individual of *P*. *glutinosus* (RH70700) was recovered within *P*. *kentucki*. When mtDNA was added to the nuclear data, the resulting phylogenetic inference largely reflected the mtDNA tree (*c*.*f*., Figure I in S4 Appendix; Figs 3 and 4), with individual RH70770 recovered as a member of a monophyletic outgroup. The discordant results with respect to individual RH70770 suggest that there is hybridization between *P*. *kentucki* and *P*. *glutinosus*, which was also documented by Highton and MacGregor [52] using allozymes.

### Initial delimitation of putative species

**Fig 5:**
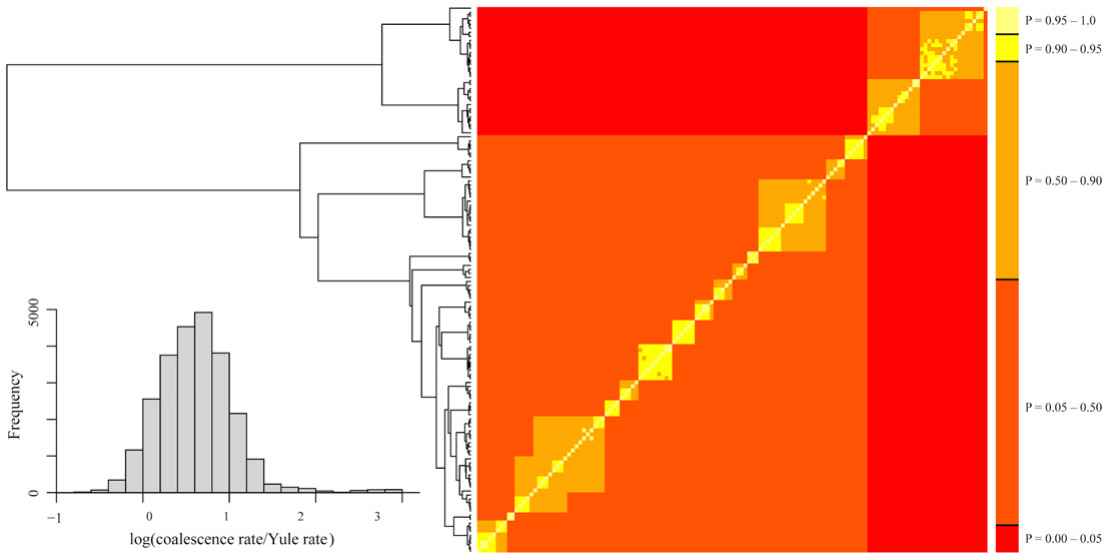
bGMYC analyses. To the left is the maximum clade credibility tree from BEAST 2 (Figs 3 and 4). The table is a sequence-by-sequence matrix, with cells colored by the posterior probability that the corresponding sequences are conspecific. Off-diagonal colors indicate uncertainty due to uncertainty in topology.

To probe our data for discrete evolutionary lineages, we used the bGMYC, Geneland, and Brownie. The bGMYC results are summarized in Fig 5. The colored matrix compares individuals, with colors corresponding to the posterior probability they are conspecific. To delimit species, it is necessary to specify a probability threshold above which individuals will be considered heterospecific. If we adopt a threshold of *P* = 0.95, two species are identified that correspond with Clade A-C vs. Clade D in our mtDNA phylogeny (Figs 3 and 4). If we use the posterior mean of the analysis as the probability threshold (*P* = 0.5), 17 species are identified. These largely correspond with statistically supported tip clades in our mtDNA phylogeny, and form geographically cohesive groups of populations (Figs 1, 3 and 4). The exception is species "P," which includes a single individual from population 40, even though four other individuals from population 40 were assigned to species "H". We lack nuclear data for species "P," and thus our validation of the 17 species delimitation (see below) included only 16 species.

**Fig 6:**
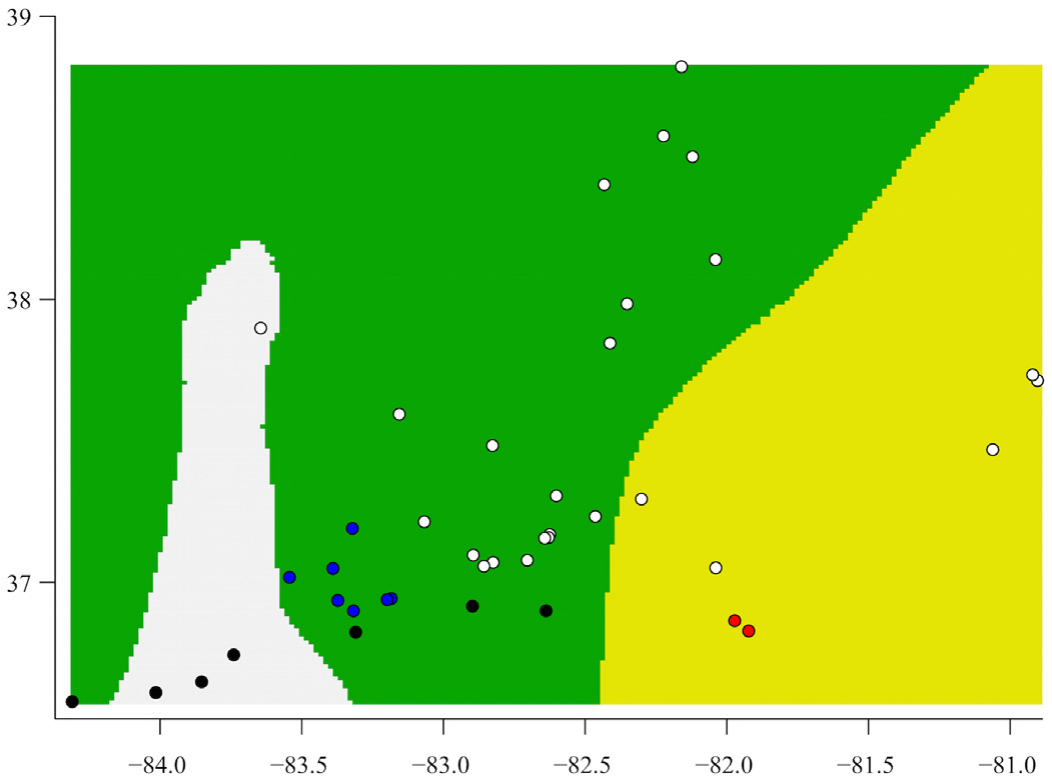
Geneland results, with grouping inferred from nuclear loci. The three colors correspond to the three groups inferred by Geneland. The dots are the collecting localities (see Fig 1), and are colored by clade: white = Clade A; blue = Clade B; red = Clade C; black = Clade D. The histogram shows the log of the ratio of the estimates rates of coalescence and the estimated Yule rates. Values above zero indicate the estimated rate of coalescence is higher.

For comparison with the bGMYC, we explored patterns in our nuclear data using Geneland. Replicates of the Geneland analysis supported recognition of three populations, including one to the east, one formed by all the central sampling localities, and one that includes three southern localities in combination with the northwestern-most sample (Fig 6). None of these groups corresponds with a mtDNA clade (Fig 3).

Finally, using Brownie we attempted to delimit species using four haploid nuclear data sets, each of which was a random sample of the larger diploid data (see Material and Methods). These were all tested with and without the addition of mtDNA. A detailed presentation of the results can be found in S5 Appendix. In brief, Brownie did not reliably delimit species. Vast differences were recovered when different data sets were used, from one to 5018 species, even though these data sets were randomly sampled from the same larger diploid data set. An analysis of the diploid data gave results that differed from all of the haploid data sets. In these analyses, the delimited species did not form contiguous geographic groups, and typically did not include all the individuals from single populations. This was true whether or not mtDNA was included. Given the inconsistency of the findings, we did not attempt to validate these putative species (see below).

### Phylogenetic inference: species trees

**Fig 7:**
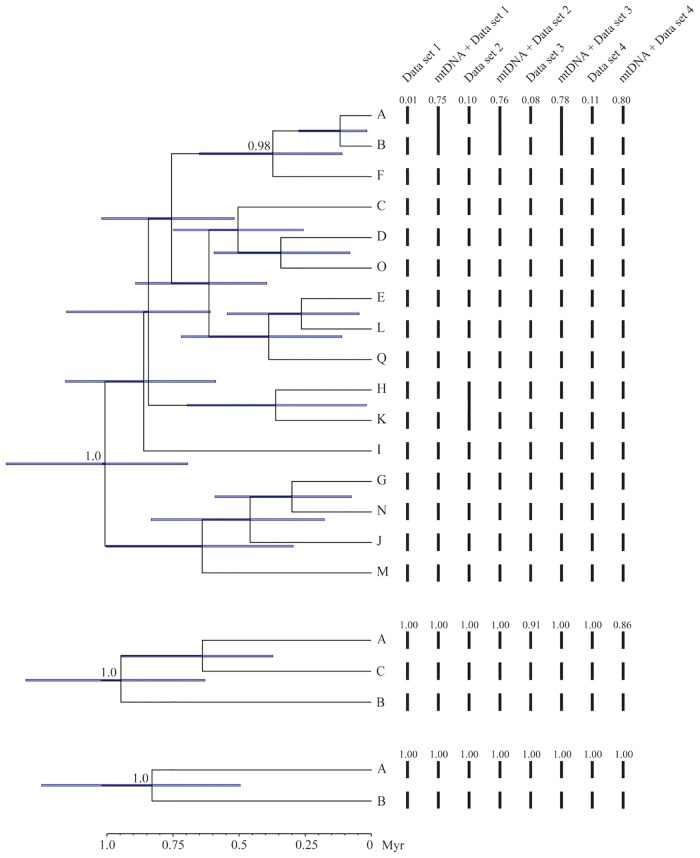
Maximum clade credibility trees from *BEAST 2, with species delimitations from BPP. Numbers at tree nodes are posterior probabilities; numbers <0.95 have been omitted. Bars at nodes represent the 95% highest posterior density for the inferred ages of nodes. To the right, bars connect putative species that were combined in the BPP analyses. (A) 16 species as delimited by bGMYC, using mtDNA data; (B) 3 species as delimited by Geneland, using the nuclear data; (4) 2 species as delimited by bGMYC, using mtDNA data. See also Fig 3 for species delimitations.

We used the results of our bGMYC (2 and 17 species) and Geneland (3 species) analyses to define *a priori* species for species tree analyses in *BEAST 2 [94]. All runs in *BEAST 2 resulted in thorough sampling of the posterior distributions (all ESS > 200). For all three phylogenies, the basal split in the tree was supported (pp = 1.00), but otherwise the trees were poorly resolved (pp < 0.95) (Fig 7). In the analysis including 16 species, only the clade including species A, B, and F was supported (pp = 0.98). All three analyses estimated the age of the MRCA at about 1 myr (range of HPDs: 0.50–1.38 myr).

### Validation of putative species

We tested for exclusive ancestry using the *gsi* with our four haploid nuclear data sets and a diploid data set. For the bGMYC delimitation of two species, no level of exclusive ancestry was detected using BFI, MLC2A, or RAG1 (S6 Appendix). GAPD and ILF3 recovered either species A, species B, or both, depending on the data set. However, the ensemble *gsi* (*egsi*), which considers all loci, detected species A and B in every data set except data set 3, which supported neither. The *egsi* values ranged from 0.14–0.24.

For data sets 1–4 and the bGMYC delimitation of 17 species, species A-C, F, I-O, and Q did not show a pattern of exclusive ancestry that was consistently different from zero at any locus. In some cases the *egsi* was significant, but this varied by data set. Species D, E, and H more consistently exhibited significant levels of exclusive ancestry, with *egsi* values that ranged between 0.20–0.25. Species G was the most consistently supported, with relatively high *egsi* values (range: 0.27–0.48). In general, the diploid data exhibited higher levels of exclusive ancestry for more loci than did the haploid data sets, with 11 of 16 *egsi* values significant (range: 0.18–0.36).

For the three species delimited using Geneland, the *gsi* provided consistent support relative to the bGMYC results. However, the same nuclear loci were used to delimit populations in Geneland, so one would expect the *gsi* to perform relatively well. Only BFI did not exhibit any patterns of exclusive ancestry. GAPD provided the strongest support, with significant *gsi* values for all three species in all data sets. Perhaps the patterns in GAPD contributed disproportionately to the population groupings recovered by Geneland. Overall, the *egsi* values, while significant, were not high (range: 0.20–0.34). When mtDNA was included, no pattern of exclusive ancestry was detected in any run. This may not be surprising, as the species delimited by Geneland do not correspond with the mtDNA phylogeny.

Finally, we used the program BPP to test the species delimited by the bGMYC (2 and 17 species) and Geneland analyses (3 species). Multiple runs of BPP produced consistent results, indicating that the MCMC chains were well mixed. For the delimitations, all four data sets were analyzed with and without mtDNA. Rannala and Yang [105] have suggested that different putative species only be considered distinct if their posterior probability exceeds a threshold such as 95% or even 99%. For the analysis of two species, the delimitation with the highest posterior probability (pp) always supported both species with pp > 0.95 (Fig 7). Similarly, with 3 species the delimitation with the highest pp always supported three species, with pp > 0.95 in 6 of 8 analyses (Fig 7). Note that these two delimitations (2 vs. 3 species) are composed of sets of OTUs that are mutually exclusive (Fig 3).

We had nuclear data for 16 of the 17 putative species delimited by the bGMYC (see above). Using the nuclear data alone, the delimitation with the highest posterior probability included either 16 (data sets 1, 3, 4) or 14 species (data set 2) (Fig 6). However, all of these delimitations had low posterior probabilities (range: <0.01 to 0.11), and no species had a pp of ≥0.95 in more than one data set. When mtDNA was included, the delimitation with the highest posterior probability included 15 or 16 species; in three of the four data sets, species A and B were combined into a single species. Posterior probabilities were substantially higher when mtDNA was included (range: 0.75–0.80), but none were ≥ 0.95.

## Discussion

**Fig 8:**
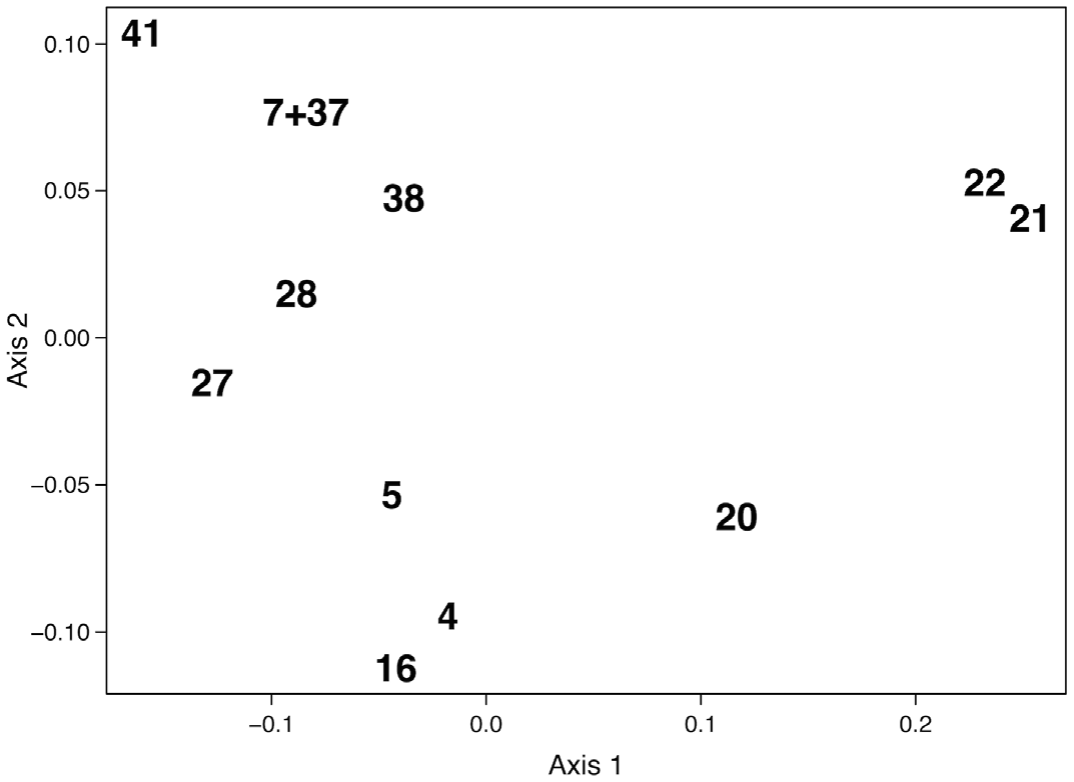
Multidimensional scaling of Nei's genetic distances. Genetic data from Highton and MacGregor (1983). Populations numbers match Fig 1.

Delimiting species when morphology is highly conserved has long challenged systematists. We studied patterns of genetic variation in the Cumberland Plateau Salamander, *P*. *kentucki*, which is a cryptic species with respect to *P*. *glutinosus*. Prior research using allozymes [52] found that *P*. *glutinosus* exhibits relatively little genetic variation where it co-occurs with *P*. *kentucki*, whereas *P*. *kentucki* possesses striking levels of variation. Despite the high level of genetic differentiation, however, populations of *P*. *kentucki* are not easily sorted into distinct, geographically cohesive groups. For example, Fig 8 presents a multidimensional scaling (MDS) analysis of Nei’s genetic distances, which was made using the allozyme data in [52]. When inter-population variation is a function of geographic distance alone, an MDS of the first two dimensions produces a clustering pattern akin to a geographical map of the populations [36,39,106,107]. In Fig 8, the populations are widely spaced and do not for distinct clusters. The exception is populations 21–22, but these populations are also the most geographically isolated (Fig 1). The high levels of genetic variation and complex population genetic structure in the allozyme data suggested to us that *P*. *kentucki* was in need of further phylogeographic evaluation.

### Phylogeographic differentiation

We sampled specimens from throughout the range of *P*. *kentucki*, and obtained sequence data from nine loci, including four mtDNA loci and five nuclear loci. According to mtDNA variation, *P*. *kentucki* is old and harbors a large amount of genetic structure, even for a relatively dispersal-limited amphibian with a small range [64,108,109]. Using Bayesian phylogenetic analyses, we recovered four divergent mitochondrial clades (Clades A-D). The basal split, which separates populations in the southwest (Clade D) from the other populations (Clades A-C), was dated at ~14.9 myr (95% HPD: 10.8–19.7 myr). Clade D is separated from Clades A-C by the upper reaches of the Cumberland River, suggesting the river could be a barrier to dispersal, though here it is not as so large as it is to the west. Similarly, north of the Cumberland, Clades A and C are separated by the upper reaches of the Kentucky River. Clades A-C have a common ancestor inferred to have existed 5.5 mya (95% HPD: 3.4–6.4 mya). Together, these clades occupy parts of the Cumberland Plateau and Valley and Ridge physiographic provinces, but the clade distributions do not follow the boundary between these provinces.

In contrast with mtDNA, nuclear loci exhibited low levels of divergence and limited phylogeographic structure (Table 2). Shared polymorphisms among populations in the nuclear data were found over broad spatial scales, a pattern that can result from retained ancestral polymorphism or introgressive hybridization [110]. Individual gene trees included few supported nodes, and did not identify geographically cohesive groups (S3 Appendix). In sum, the nuclear loci in this study provided low levels phylogeographic variation, in striking contrast with mtDNA. All of the nuclear loci used in our study, except for BFI, were used by Fisher-Reid and Wiens [70] in a phylogenetic analysis of relationships within *Plethodon*, with some success. We thus reasoned that these loci had a good chance of diagnosing clearly demarcated species within *P*. *kentucki*.

The low variation and lack of monophyly in our nuclear data are not necessarily fatal for species delimitation and species tree inference, as the multispecies coalescent accounts for stochasticity in the coalescent process [25,111–113]. Introgressive hybridization, however, is not modeled by most methods, and can be problematic [114–117]. One important consequence of introgression is species tree "compression" [115], whereby divergence times are severely underestimated. This occurs because the multispecies coalescent assumes all gene tree discordance is a consequence of incomplete lineage sorting, which requires speciation events to follow coalescent events [117,118]. In *P*. *kentucki*, we may have observed species tree compression as all three species trees estimated the root of the phylogeny at around 1.0 mya (Fig 6), whereas our mtDNA estimate was 14.9 mya (Fig 3). An alternative explanation is that *P*. *kentucki* is not deeply differentiated, but rather mtDNA is maintaining a signal reflective of ancient divergence events not recorded in the nuclear genome. This interpretation, however, conflicts with published allozyme data, which revealed high levels of diversity [52].

### Species delimitation and validation

The problem of reconstructing species boundaries from genetic data is demanding. Molecular approaches to species delimitation, which hold great promise for diagnosing independent metapopulation-level evolutionary lineages, have been undergoing rapid development for the last 10–15 years [5]. This has yielded a wide array of methods, many of which incorporate advances in coalescent theory and the multispecies coalescent. Nonetheless, species formation is not so tidy as the word "speciation" implies, but is a time-extended process with complex dynamics through space and time [3]. Accordingly, the parameter space relevant for species delimitation is extraordinarily complex. By contrast, all methods of species delimitation make a number of simplifying assumptions that may cause them to fail under some real world circumstances [31,54]. In *P*. *kentucki*, we adopted a two-step approach to species delimitation: first, we delimited putative species using the bGMYC (mtDNA), Geneland (nuclear DNA), and Brownie (nuclear DNA and mtDNA); second, we validated these putative species using the *gsi* and BPP. *A priori*, we assumed (conservatively) that strongly supported species would be recovered by diverse methods [11,31]. Because our mtDNA phylogeny was much more resolved than any of our nuclear trees, we first used the bGMYC. When we used a probability of conspecificity threshold of *P* = 0.95, two species that corresponded with Clades A-C and Clade D on our mtDNA tree were identified (Figs 3 and 4). For this delimitation, ensemble *gsi* (*egsi*) values ranged from 0.19–0.23, and BPP strongly supported the existence of both species. When we explored a threshold of *P* = 0.5, 17 species were delimited, 16 of which corresponded with geographically cohesive groups of populations. However, support for these putative species was weak. *Egsi* results were inconsistent (S6 Appendix), and BPP did not strongly support any delimitation or any single species.

For comparison with our bGMYC results, we used Geneland to identify population clusters in the nuclear data. Three groups were recovered, none of which matched any of the mtDNA clades (Figs 3, 4 and 6). Thus, the putative species identified using the bGMYC and Geneland are mutually exclusive. Ensemble *gsi* values calculated from the nuclear data supported the exclusivity of these species (though there is an element of circularity, as the same nuclear data were used to define the putative species in Geneland). In BPP, the delimitation with the highest posterior probability included all three species in all analyses.

Finally, we analyzed our data using a nonparametric method that recovers species boundaries by minimizing interspecific congruence while maximizing intraspecific incongruence [59], as implemented in the program Brownie. Brownie produced erratic results, with the number of delimited species ranging from 1 to 5018. Moreover, the delimited species did not form contiguous geographic groups, and did not include all the individuals from single populations, whether or not mtDNA was included. In our analyses, we used four haploid data sets each randomly drawn from a diploid data set, and each data set produced different results. This suggests caution is warranted when a single random sample of alleles is used in Brownie and other programs [11,98,119].

### Taxonomic Implications

While recent advances in the use of genetic data to diagnose cryptic evolutionary lineages are truly exciting, they are also under active development [5,118,120–122]. In particular, most methods do not account for gene flow, isolation by distance, and population fragmentation [28,31,118,121,123], all of which are common in many natural systems. High levels of population structure, and perhaps hybridization with *P*. *glutinosus*, characterizes *P*. *kentucki*. Thus, *P*. *kentucki* is likely a difficult test case for species delimitation methods. Wakeley [124] has described the genealogical pattern resulting from a fragmented metapopulation as possessing two phases: the scattering phase, which is relatively short and characterized by rapid coalescence within demes, and the collecting phase, in which each deme is its own lineage. Relative to the scattering phase, coalescence between demes is a time-extended process. Consequently, population structure will produce clustering patterns similar to those expected under the GMYC because lineages residing in the same deme coalesce more rapidly on average than those in different demes. The GMYC, and perhaps Brownie, risk diagnosing the scattering phase as the coalescent process and the collecting phase as the Yule process [125–127]. For the bGMYC to be effective, the rate of branching for the coalescent process should be much higher than the rate of branching under a Yule process. When this is not true, the model is in an area of parameter space that may not provide reliable results. We evaluated this assumption of the bGMYC by examining the distribution of the log of the ratio of the coalescence rate to the Yule rate (Fig 5). The mass of the distribution is between zero and one, suggesting that the rate of coalescence is higher than Yule rate, but not appreciably so. In addition, several of the estimates are negative, which occurs when the estimated coalescence rate is lower than the estimated Yule rate. Overall, these results indicate that the bGMYC may not be effective at identifying species boundaries in our data. In addition, a recent study by Dellicour et al. [54] suggests that GMYC models suffer especially poor performance in data sets comprised of 1–2 species, which could be the situation in *P*. *kentucki*.

In this study we validated putative species using the multispecies coalescent in BBP. The advantages of this program are that it takes sequence data (not gene trees) as input, accommodates uncertainties in the topologies and branch lengths of inferred gene trees, and accounts for ancestral polymorphism and incomplete lineage sorting. On the downside, it assumes neutral, clock-like evolution at each locus and a simple mutation model[26], and thus may not produce accurate representations of gene tree posteriors when divergence levels are high, as with our mtDNA data. BPP also assumes no gene flow between populations, though simulations suggest that low levels of gene flow may not be problematic [128]. Comparison of our mtDNA gene tree and our species trees suggests that introgressive hybridization with *P*. *glutinosus* may have impacted the species tree in *P*. *kentucki*. Whether gene flow among putative lineages of *P*. *kentucki* was sufficient to confound inference in BPP awaits further study.

Given the high levels of population structure, and the specter of introgressive hybridization, *P*. *kentucki* may be a worst-case scenario for many recently developed methods. In this study we obtained supported delimitations of two and three putative species that were mutually exclusive. This highlights the critical importance of identifying sets of putative species, as an incorrect delimitation can receive strong statistical validation. Our two species delimitation would seem reasonable if the three species delimitation had not been tested. As a partial solution to discordant results, some researchers advocate that molecular taxonomy simultaneously adopt several approaches [11,31], as we've done here. Given the conflicting results reported in this study, we do not currently advocate taxonomic changes. A weakness in our study is that the five nuclear loci employed all exhibited very low levels of variation, a surprise given the high levels of allozyme differentiation previously recorded [52] and the high levels of mtDNA variation we documented. Future work will revisit the problem of geographically structured genetic variation and species delimitation in *P*. *kentucki* using next generation sequence data.

## Supporting Information

S1 Appendix Detailed sampling information. Table A.
(PDF)

S2 Appendix Multidivtime analyses. Methods, Table B, Figures A, B.
(PDF)

S3 Appendix Gene trees for nuclear loci. Figures C-G.
(PDF)

S4 Appendix Concatenated analyses using RAxML, including a phylogeny of the nuclear loci, and a phylogeny with both nuclear and mtDNA loci. Figures H-I.
(PDF)

S5 Appendix Brownie. Results, Tables C-H, Figures J-K.
(PDF)

S6 Appendix Genealogical sorting index results. Table I.
(PDF)
