# Supplementary material for: Multilocus Phylogeography and Species Delimitation in the Cumberland Plateau Salamander, Plethodon kentucki: Incongruence among Data Sets and Methods
Source: PLoS One. 2016 Mar 14;11(3):e0150022. doi: 10.1371/journal.pone.0150022 (PMC4790894; doi:10.1371/journal.pone.0150022)
Supplement: S1 Appendix — Table A. (PDF) [file pone.0150022.s001.pdf]

Table A. Sampling information. Population numbers correspond to Figure 1 and are used throughout the text. The "Complete" data set has no missing data for any locus (see text), and is identified using check marks. MtDNA haplotypes were uploaded to GenBank as an alignment; check marks indentify the loci included for each individual. All specimens are *Plethodon kentucki*, except for the last four specimens, which are *Plethodon glutinosus* and were used as outgroups some analyses.

Pop	Specimen	Locality	County	State	Latitude	Longitude	Complete	mtDNA	CytB	ND2	tRNA-trp	tRNA-ala	BFI	GAPD	MLC2A	ILF3	RAG-1
1	SRK3022	Point Pleasant	Mason	WV	38.8196	-82.1611	–	KU840930	✔	✔	✔	✔	KU841067	KU841144	KU841294	–	–
1	SRK3023	Point Pleasant	Mason	WV	38.8196	-82.1611	–	KU840931	✔	✔	✔	✔	KU841068	KU841145	KU841295	–	–
2	RH65589	Henderson	Mason	WV	38.8219	-82.1558	✔	KU840999	✔	✔	✔	✔	KU841110	KU841186	KU841333	KU841247	KU841409
2	RH65590	Henderson	Mason	WV	38.8219	-82.1558	✔	KU841000	✔	✔	✔	✔	KU841111	KU841187	KU841334	KU841256	KU841410
2	RH65591	Henderson	Mason	WV	38.8219	-82.1558	–	KU841001	✔	–	–	–	–	–	KU841335	–	–
3	RH65666	Union Ridge Road	Cabell	WV	38.5772	-82.2275	✔	KU841002	✔	✔	✔	✔	KU841112	KU841188	KU841336	KU841266	KU841411
3	RH65667	Union Ridge Road	Cabell	WV	38.5772	-82.2275	✔	KU841003	✔	✔	✔	✔	KU841113	KU841189	KU841337	KU841261	KU841412
3	RH65668	Union Ridge Road	Cabell	WV	38.5772	-82.2275	–	KU841004	✔	–	–	–	–	–	KU841338	–	–
4	RH53378	Huntington	Cabell	WV	38.4019	-82.4367	✔	KU840956	✔	✔	✔	✔	KU841080	KU841156	KU841305	KU841222	KU841379
4	RH53379	Huntington	Cabell	WV	38.4019	-82.4367	✔	KU840957	✔	✔	✔	✔	KU841081	KU841157	KU841306	KU841270	KU841380
5	RH52628	3.5 km SSW of Virgie	Pike	KY	37.3064	-82.5972	✔	KU840938	✔	✔	✔	✔	KU841072	KU841149	KU841299	KU841264	KU841372
5	RH52629	3.5 km SSW of Virgie	Pike	KY	37.3064	-82.5972	✔	KU840939	✔	✔	✔	✔	KU841073	KU841150	KU841300	KU841263	KU841373
5	RH52631	3.5 km SSW of Virgie	Pike	KY	37.3064	-82.5972	–	KU840940	✔	–	–	–	–	–	–	–	–
5	RH52632	3.5 km SSW of Virgie	Pike	KY	37.3064	-82.5972	–	KU840941	✔	–	–	–	–	–	–	–	–
5	RH54238	3.5 km SSW of Virgie	Pike	KY	37.3064	-82.5972	–	KU840962	✔	–	–	–	–	–	–	–	–
6	RH79575	North Fork Road	Wise	VA	37.0817	-82.7036	✔	KU841060	✔	✔	✔	✔	KU841141	KU841217	KU841364	KU841225	KU841438
7	RH53307	1.5 km NE of Pound Gap	Letcher	KY	37.1658	-82.6250	✔	KU840953	✔	✔	✔	✔	KU841078	KU841155	KU841304	KU841244	KU841378
7	RH53308	1.5 km NE of Pound Gap	Letcher	KY	37.1658	-82.6250	–	KU840954	✔	–	–	–	KU841079	–	–	–	–
7	RH53309	1.5 km NE of Pound Gap	Letcher	KY	37.1658	-82.6250	–	KU840955	✔	–	–	–	–	–	–	–	–
7	RH54812	1.5 km NE of Pound Gap	Letcher	KY	37.1658	-82.6250	✔	KU840963	✔	✔	✔	✔	KU841085	KU841161	KU841308	KU841274	KU841384
7	RH54813	1.5 km NE of Pound Gap	Letcher	KY	37.1658	-82.6250	–	KU840964	✔	✔	–	–	–	–	–	–	–
8	RH55951	Pound Mountain Tower	Wise	VA	37.1528	-82.6428	✔	KU840967	✔	✔	✔	✔	KU841088	KU841164	KU841311	KU841268	KU841387
9	RH78206	Pound Gap	Letcher	KY	37.1553	-82.6336	✔	KU841048	✔	✔	✔	✔	KU841135	KU841210	KU841359	KU841254	KU841432
9	RH78479	Pound Gap	Letcher	KY	37.1553	-82.6336	✔	KU841056	✔	✔	✔	–	KU841140	KU841215	KU841363	KU841259	KU841437
10	RH60245	Oscaloosa	Letcher	KY	37.0967	-82.8908	✔	KU840981	✔	✔	✔	✔	KU841097	KU841173	KU841320	KU841221	KU841396
10	RH60246	Oscaloosa	Letcher	KY	37.0967	-82.8908	✔	KU840982	✔	✔	✔	✔	KU841098	KU841174	KU841321	KU841223	KU841397
11	RH77587	3 miles E Scuttlehole Gap	Letcher	KY	37.0667	-82.8250	✔	KU841023	✔	✔	✔	✔	KU841124	KU841199	KU841347	KU841235	KU841421
11	RH77588	3 miles E Scuttlehole Gap	Letcher	KY	37.0667	-82.8250	✔	KU841024	✔	✔	✔	✔	KU841125	KU841200	KU841348	KU841265	KU841422
11	RH77589	3 miles E Scuttlehole Gap	Letcher	KY	37.0667	-82.8250	–	KU841025	✔	✔	–	–	–	–	–	–	–
11	RH78157	4 miles E Scuttlehole Gap	Letcher	KY	37.0667	-82.8250	–	KU841038	✔	–	–	–	–	–	KU841355	–	–
11	RH78158	5 miles E Scuttlehole Gap	Letcher	KY	37.0667	-82.8250	–	KU841039	✔	–	–	–	–	–	–	–	–
12	RH77616	Scuttlehole Gap	Letcher	KY	37.0536	-82.8575	✔	KU841036	✔	✔	✔	✔	KU841130	KU841205	KU841353	KU841224	KU841427
12	RH77617	Scuttlehole Gap	Letcher	KY	37.0536	-82.8575	✔	KU841037	✔	✔	✔	✔	KU841131	KU841206	KU841354	KU841236	KU841428
12	RH78481	Scuttlehole Gap	Letcher	KY	37.0536	-82.8575	–	KU841057	✔	–	–	–	–	–	–	–	–
13	RH78215	Near Flat Gap	Dickenson	VA	37.2333	-82.4658	✔	KU841049	✔	✔	✔	✔	KU841136	KU841211	KU841360	KU841283	KU841433
13	RH78216	Near Flat Gap	Dickenson	VA	37.2333	-82.4658	✔	KU841050	✔	✔	✔	✔	KU841137	KU841212	KU841361	KU841285	KU841434
13	RH78490	Near Flat Gap	Dickenson	VA	37.2333	-82.4658	–	KU841058	✔	–	–	–	–	KU841216	–	–	–
13	RH78491	Near Flat Gap	Dickenson	VA	37.2333	-82.4658	–	KU841059	✔	–	–	–	–	–	–	–	–
14	RH77591	West Garrett	Floyd	KY	37.4833	-82.8311	✔	KU841026	✔	✔	✔	✔	KU841126	KU841201	KU841349	KU841231	KU841423
14	RH77592	West Garrett	Floyd	KY	37.4833	-82.8311	–	KU841027	✔	–	–	–	–	–	–	–	–
14	RH77593	West Garrett	Floyd	KY	37.4833	-82.8311	–	KU841028	✔	–	–	–	–	–	–	–	–
14	RH77595	West Garrett	Floyd	KY	37.4833	-82.8311	✔	KU841029	✔	✔	✔	✔	KU841127	KU841202	KU841350	KU841257	KU841424
14	RH77596	West Garrett	Floyd	KY	37.4833	-82.8311	–	KU841030	✔	–	–	–	–	–	–	–	–
15	SRK3197	Kanawha State Forest Site 6	Kanawha	WV	38.1411	-82.0439	✔	KU840932	✔	✔	✔	✔	KU841069	KU841146	KU841296	KU841262	KU841369
16	RH55651	Cabwaylingo State Forest	Wayne	WV	37.9906	-82.3503	✔	KU840965	✔	✔	✔	✔	KU841086	KU841162	KU841309	KU841260	KU841385
16	RH55652	Cabwaylingo State Forest	Wayne	WV	37.9906	-82.3503	✔	KU840966	✔	✔	✔	✔	KU841087	KU841163	KU841310	KU841258	KU841386
16	RH70763	Cabwaylingo State Forest	Wayne	WV	37.9906	-82.3503	–	KU841017	✔	–	–	–	–	–	–	–	–
17	SRK3203	Mill Cr. Wildlife Management Area	Cabell	WV	38.4951	-82.1151	✔	KU841062	✔	✔	✔	✔	KU841142	KU841218	KU841367	KU841291	KU841440
17	SRK3204	Mill Cr. Wildlife Management Area	Cabell	WV	38.4951	-82.1151	–	KU841063	✔	✔	✔	✔	–	KU841219	KU841368	KU841292	KU841441
18	SRK3199	Beech Fork State Park	Cabell	WV	38.3138	-82.3387	–	KU841061	✔	✔	✔	✔	–	–	KU841366	KU841290	KU841439

Pop	Specimen	Locality	County	State	Latitude	Longitude	Complete	mtDNA	CytB	ND2	tRNA-trp	tRNA-ala	BFI	GAPD	MLC2A	ILF3	RAG-1
19	RH60231	Collins Creek	Martin	KY	37.8411	-82.4117	✔	KU840979	✔	✔	✔	✔	KU841095	KU841171	KU841318	KU841249	KU841394
19	RH60233	Collins Creek	Martin	KY	37.8411	-82.4117	✔	KU840980	✔	✔	✔	✔	KU841096	KU841172	KU841319	KU841240	KU841395
20	RH60215	Breaks Interstate Park	Dickenson	VA	37.2942	-82.3050	✔	KU840973	✔	✔	✔	✔	KU841093	KU841169	KU841316	KU841251	KU841392
20	RH60216	Breaks Interstate Park	Dickenson	VA	37.2942	-82.3050	✔	KU840974	✔	✔	✔	✔	KU841094	KU841170	KU841317	KU841277	KU841393
20	RH60217	Breaks Interstate Park	Dickenson	VA	37.2942	-82.3050	–	KU840975	✔	–	–	–	–	–	–	–	–
20	RH60219	Breaks Interstate Park	Dickenson	VA	37.2942	-82.3050	–	KU840976	✔	–	–	–	–	–	–	–	–
20	RH60220	Breaks Interstate Park	Dickenson	VA	37.2942	-82.3050	–	KU840977	✔	–	–	–	–	–	–	–	–
20	RH60223	Breaks Interstate Park	Dickenson	VA	37.2942	-82.3050	–	KU840978	✔	–	–	–	–	–	–	–	–
21	RH54111	Hinton (S)	Raleigh	WV	37.7142	-80.8950	✔	KU840958	✔	✔	✔	✔	KU841082	KU841158	KU841307	KU841282	KU841381
21	RH57300	Hinton (S)	Raleigh	WV	37.7142	-80.8950	✔	KU840972	✔	✔	✔	✔	KU841092	KU841168	KU841315	KU841281	KU841391
22	RH62899	Hinton (N)	Raleigh	WV	37.7339	-80.9167	✔	KU840990	✔	✔	✔	✔	KU841103	KU841179	KU841326	KU841272	KU841402
22	RH62902	Hinton (N)	Raleigh	WV	37.7339	-80.9167	✔	KU840991	✔	✔	✔	✔	KU841104	KU841180	KU841327	KU841241	KU841403
22	RH62903	Hinton (N)	Raleigh	WV	37.7339	-80.9167	–	–	–	–	–	–	–	–	KU841365	–	–
23	RH69455	Brush Creek Falls	Mercer	WV	37.4647	-81.0619	✔	KU841010	✔	✔	✔	✔	KU841116	KU841192	KU841341	KU841275	KU841415
24	RH69834	Big A Mountain	Buchanan	VA	37.0514	-82.0425	✔	KU841011	✔	✔	✔	✔	KU841117	KU841193	KU841342	KU841250	KU841416
24	RH69835	Big A Mountain	Buchanan	VA	37.0514	-82.0425	–	KU841012	✔	–	–	–	–	–	–	–	–
24	RH69838	Big A Mountain	Buchanan	VA	37.0514	-82.0425	✔	KU841013	✔	✔	✔	–	KU841118	KU841194	KU841343	KU841273	KU841417
24	RH69839	Big A Mountain	Buchanan	VA	37.0514	-82.0425	–	KU841014	✔	–	–	–	–	–	–	–	–
24	RH69840	Big A Mountain	Buchanan	VA	37.0514	-82.0425	–	KU841015	✔	–	–	–	–	–	–	–	–
25	RH60257	0.7 miles N. of Sassafras	Knott	KY	37.2300	-83.0576	✔	KU840984	✔	✔	–	–	KU841100	KU841176	KU841323	KU841229	KU841399
26	RH77576	Big Sourwood Branch Gauge	Breathitt	KY	37.5903	-83.1556	–	KU841020	✔	✔	✔	✔	KU841122	KU841197	KU841345	KU841288	KU841419
26	RH77577	Big Sourwood Branch Gauge	Breathitt	KY	37.5903	-83.1556	✔	KU841021	✔	✔	✔	✔	KU841123	KU841198	KU841346	KU841255	KU841420
26	RH77578	Big Sourwood Branch Gauge	Breathitt	KY	37.5903	-83.1556	–	KU841022	✔	–	–	–	–	–	–	–	–
27	RH57058	Log Mtn; 5.5 km SSW of Frenchburg	Menifee	KY	37.9033	-83.6450	✔	KU840969	✔	✔	✔	✔	KU841090	KU841166	KU841313	KU841271	KU841389
27	RH57064	Log Mtn; 5.5 km SSW of Frenchburg	Menifee	KY	37.9033	-83.6450	✔	KU840970	✔	✔	✔	✔	KU841091	KU841167	KU841314	KU841248	KU841390
27	RH57065	Log Mtn; 5.5 km SSW of Frenchburg	Menifee	KY	37.9033	-83.6450	–	KU840971	✔	–	–	–	–	–	–	–	–
28	RH52677	Pine Mountain	Harlan	KY	36.9336	-83.1975	✔	KU840942	✔	✔	✔	✔	KU841074	KU841151	KU841301	KU841239	KU841374
28	RH52678	Pine Mountain	Harlan	KY	36.9336	-83.1975	–	KU840943	✔	–	–	–	–	–	–	–	–
28	RH52682	Pine Mountain	Harlan	KY	36.9336	-83.1975	–	KU840944	✔	–	–	–	–	–	–	–	–
28	RH52683	Pine Mountain	Harlan	KY	36.9336	-83.1975	✔	KU840945	✔	✔	✔	✔	KU841075	KU841152	KU841302	KU841242	KU841375
28	RH52688	Pine Mountain	Harlan	KY	36.9336	-83.1975	–	KU840946	✔	–	–	–	–	–	–	–	–
29	RH56194	Pine Mt. Set School	Harlan	KY	36.9378	-83.1733	✔	KU840968	✔	✔	✔	✔	KU841089	KU841165	KU841312	KU841228	KU841388
30	RH60255	Asher	Leslie	KY	37.0486	-83.3903	✔	KU840983	✔	✔	✔	✔	KU841099	KU841175	KU841322	KU841253	KU841398
31	RH78178	Upper Bear Creek	Clay	KY	37.0181	-83.5389	✔	KU841040	✔	✔	✔	✔	KU841132	KU841207	KU841356	KU841232	KU841429
31	RH78179	Upper Bear Creek	Clay	KY	37.0181	-83.5389	✔	KU841041	✔	✔	✔	✔	KU841133	KU841208	KU841357	KU841267	KU841430
31	RH78180	Upper Bear Creek	Clay	KY	37.0181	-83.5389	–	KU841042	✔	–	–	–	–	–	–	–	–
31	RH78181	Upper Bear Creek	Clay	KY	37.0181	-83.5389	–	KU841043	✔	–	–	–	–	–	–	–	–
31	RH78182	Upper Bear Creek	Clay	KY	37.0181	-83.5389	–	KU841044	✔	–	–	–	–	–	–	–	–
32	RH78217	Pine Mountain	Harlan	KY	36.8994	-83.3158	✔	KU841051	✔	✔	✔	✔	KU841138	KU841213	KU841362	KU841233	KU841435
32	RH78218	Pine Mountain	Harlan	KY	36.8994	-83.3158	✔	KU841052	✔	✔	✔	✔	KU841139	KU841214	KU863661	KU841278	KU841436
32	RH78219	Pine Mountain	Harlan	KY	36.8994	-83.3158	–	KU841053	✔	✔	–	–	–	–	–	–	–
32	RH78220	Pine Mountain	Harlan	KY	36.8994	-83.3158	–	KU841054	✔	–	–	–	–	–	–	–	–
32	RH78221	Pine Mountain	Harlan	KY	36.8994	-83.3158	–	KU841055	✔	–	–	–	–	–	–	–	–
33	RH78198	Cawood Branch	Leslie	KY	36.9361	-83.3717	✔	KU841047	✔	✔	✔	✔	KU841134	KU841209	KU841358	KU841284	KU841431
34	RH61393	Wootin	Leslie	KY	37.1869	-83.3208	✔	KU840985	✔	✔	✔	✔	KU841101	KU841177	KU841324	KU841238	KU841400
34	RH61396	Wootin	Leslie	KY	37.1869	-83.3208	✔	KU840986	✔	✔	✔	–	KU841102	KU841178	KU841325	KU841237	KU841401
34	RH61397	Wootin	Leslie	KY	37.1869	-83.3208	–	KU840987	✔	–	–	–	–	–	–	–	–
34	RH61401	Wootin	Leslie	KY	37.1869	-83.3208	–	KU840988	✔	–	–	–	–	–	–	–	–
34	RH61402	Wootin	Leslie	KY	37.1869	-83.3208	–	KU840989	✔	–	–	–	–	–	–	–	–
35	RH69433	Hayters Gap	Washington	VA	36.8244	-81.9231	✔	KU841005	✔	✔	✔	✔	KU841114	KU841190	KU841339	KU841234	KU841413
35	RH69434	Hayters Gap	Washington	VA	36.8244	-81.9231	–	KU841006	✔	✔	✔	✔	KU841115	KU841191	KU841340	KU841276	KU841414
35	RH69435	Hayters Gap	Washington	VA	36.8244	-81.9231	–	KU841007	✔	–	–	–	–	–	–	–	–
35	RH69436	Hayters Gap	Washington	VA	36.8244	-81.9231	–	KU841008	✔	–	–	–	–	–	–	–	–
35	RH69437	Hayters Gap	Washington	VA	36.8244	-81.9231	–	KU841009	✔	–	–	–	–	–	–	–	–

Pop	Specimen	Locality	County	State	Latitude	Longitude	Complete	mtDNA	CytB	ND2	tRNA-trp	tRNA-ala	BFI	GAPD	MLC2A	ILF3	RAG-1
36	RH70699	Near Middle Knob	Washington	VA	36.8689	-81.9722	✔	KU841016	✔	✔	✔	✔	KU841119	KU841195	KU841344	KU841279	KU841418
37	RH51859	Big Black Mountain	Harlan	KY	36.9175	-82.9011	✔	KU840933	✔	✔	✔	✔	KU841070	KU841147	KU841297	KU841226	KU841370
37	RH51866	Big Black Mountain	Harlan	KY	36.9175	-82.9011	✔	KU840934	✔	✔	✔	✔	KU841071	KU841148	KU841298	KU841227	KU841371
37	RH51868	Big Black Mountain	Harlan	KY	36.9175	-82.9011	–	KU840935	✔	✔	–	–	–	–	–	–	–
37	RH51870	Big Black Mountain	Harlan	KY	36.9175	-82.9011	–	KU840936	✔	–	–	–	–	–	–	–	–
37	RH51871	Big Black Mountain	Harlan	KY	36.9175	-82.9011	–	KU840937	✔	–	–	–	–	–	–	–	–
38	RH52769	High Knob	Wise	VA	36.8950	-82.6328	✔	KU840947	✔	✔	✔	✔	KU841076	KU841153	KU841303	KU841269	KU841376
38	RH52770	High Knob	Wise	VA	36.8950	-82.6328	–	KU840948	–	✔	✔	–	–	–	–	–	–
38	RH52779	High Knob	Wise	VA	36.8950	-82.6328	–	KU840949	✔	–	–	–	–	–	–	–	–
38	RH52780	High Knob	Wise	VA	36.8950	-82.6328	✔	KU840950	✔	✔	✔	✔	KU841077	KU841154	KU863658	KU841252	KU841377
38	RH52783	High Knob	Wise	VA	36.8950	-82.6328	–	KU840951	✔	–	–	–	–	–	–	–	–
38	RH52784	High Knob	Wise	VA	36.8950	-82.6328	–	KU840952	✔	–	–	–	–	–	–	–	–
38	RH74950	High Knob	Wise	VA	36.8950	-82.6328	–	–	–	AY875027	AY875027	AY875027	–	–	–	–	–
39	RH64610	Harlan	Harlan	KY	36.8278	-83.3094	✔	KU840993	✔	✔	–	–	KU841106	KU841182	KU841329	KU841220	KU841405
39	RH64611	Harlan	Harlan	KY	36.8278	-83.3094	–	KU840994	✔	✔	✔	✔	KU841107	KU841183	KU841330	–	KU841406
40	RH54117	Bear Wallow Gap	Bell	KY	36.7439	-83.7442	✔	KU840959	✔	✔	✔	✔	KU841083	KU841159	KU863659	KU841246	KU841382
40	RH54118	Bear Wallow Gap	Bell	KY	36.7439	-83.7442	✔	KU840960	✔	✔	✔	✔	KU841084	KU841160	KU863660	KU841245	KU841383
40	RH54119	Bear Wallow Gap	Bell	KY	36.7439	-83.7442	–	KU840961	✔	–	–	–	–	–	–	–	–
40	RH78194	Bear Wallow Gap	Bell	KY	36.7439	-83.7442	–	KU841045	✔	–	–	–	–	–	–	–	–
40	RH78195	Bear Wallow Gap	Bell	KY	36.7439	-83.7442	–	KU841046	✔	–	–	–	–	–	–	–	–
41	RH64622	Log Mtn; 5.5 km SSW of Frenchburg	Bell	KY	36.6489	-83.8494	✔	KU840995	✔	✔	✔	✔	KU841108	KU841184	KU841331	KU841230	KU841407
41	RH64623	Log Mtn; 5.5 km SSW of Frenchburg	Bell	KY	36.6489	-83.8494	✔	KU840996	✔	✔	✔	✔	KU841109	KU841185	KU841332	KU841280	KU841408
41	RH64625	Log Mtn; 5.5 km SSW of Frenchburg	Bell	KY	36.6489	-83.8494	–	KU840997	✔	✔	✔	–	–	–	–	–	–
41	RH64627	Log Mtn; 5.5 km SSW of Frenchburg	Bell	KY	36.6489	-83.8494	–	KU840998	✔	–	–	–	–	–	–	–	–
41	RH66693	Log Mtn; 5.5 km SSW of Frenchburg	Bell	KY	36.6481	-83.8494	–	–	DQ994948	–	–	–	–	JN798240	JN798331	JN798279	DQ995033
41	RH75685	Log Mtn; 5.5 km SSW of Frenchburg	Bell	KY	36.6481	-83.8494	–	KU841018	✔	✔	✔	✔	KU841120	KU841196	–	–	–
41	RH75690	Log Mtn; 5.5 km SSW of Frenchburg	Bell	KY	36.6481	-83.8494	–	KU841019	–	✔	✔	✔	KU841121	–	–	–	–
42	RH63677	Ketchen	Scott	TN	36.5764	-84.3072	✔	KU840992	✔	✔	✔	✔	KU841105	KU841181	KU841328	KU841287	KU841404
43	RH77607	Limestone Cave	Whitley	KY	36.6108	-84.0189	✔	KU841031	✔	✔	✔	✔	KU841128	KU841203	KU841351	KU841243	KU841425
43	RH77608	Limestone Cave	Whitley	KY	36.6108	-84.0189	✔	KU841032	✔	✔	✔	✔	KU841129	KU841204	KU841352	KU841289	KU841426
43	RH77609	Limestone Cave	Whitley	KY	36.6108	-84.0189	–	KU841033	✔	✔	✔	–	–	–	–	–	–
43	RH77610	Limestone Cave	Whitley	KY	36.6108	-84.0189	–	KU841034	✔	–	–	–	–	–	–	–	–
43	RH77611	Limestone Cave	Whitley	KY	36.6108	-84.0189	–	KU841035	✔	–	–	–	–	–	–	–	–
–	RH70700	Near Middle Knob	Washington	VA	36.8689	-81.9722	–	KU840926	✔	✔	✔	✔	–	KU841143	–	–	–
–	RH70701	Near Middle Knob	Washington	VA	36.8689	-81.9722	–	KU840927	✔	✔	✔	✔	KU841064	–	KU841293	–	–
–	RH70702	Near Middle Knob	Washington	VA	36.8689	-81.9722	–	KU840928	✔	✔	✔	✔	KU841065	–	–	–	–
–	RH70703	Near Middle Knob	Washington	VA	36.8689	-81.9722	–	KU840929	✔	✔	✔	✔	KU841066	–	–	–	–
