# Supplementary material for: Multilocus Phylogeography and Species Delimitation in the Cumberland Plateau Salamander, Plethodon kentucki: Incongruence among Data Sets and Methods
Source: PLoS One. 2016 Mar 14;11(3):e0150022. doi: 10.1371/journal.pone.0150022 (PMC4790894; doi:10.1371/journal.pone.0150022)
Supplement: S2 Appendix — Methods, Table B, Figures A, B. (PDF) [file pone.0150022.s002.pdf]

Appendix S2

We estimated rates of molecular evolution using Bayesian relaxed-clock dating with PAML 4.1 [1] and the set of programs in Multidistribute [2-4]. Methods are presented in the text, but here we expand upon the locus BFI, which had few sequences available outside of *P. kentucki*. First, we estimated the rate of evolution using a three-taxon statement, with *P. wehrlei* as the outgroup and *P. glutinous* + *P. kentucki* as the ingroup. This is described in the main text. Because this estimate of molecular evolution was derived from limited sampling, we also estimated the rate of evolution for BFI using a phylogeny of the Salamandridae from Zhang et al.[5] in combination with sequence data on salamandrids from Sequeira et al.[6]. The outgroup was designated as *Salamandrina terdigitata*, and the ingroup included nine taxa from eight genera (Fig. B). The upper and lower limits on node ages were set based on the BEAST 2 analyses of Zhang et al. [5], including both their primary and additional calibration points (see [5] for details). For all loci, MCMC analyses included 100,000 generations of burn-in. Thereafter, the Markov chain was sampled every 100 generations until 10,000 samples were collected.

When BFI was calibrated using *Plethodon*, the estimated rate of evolution was 0.036%/myr, which is an intermediate rate among the nuclear loci. When BFI was dated using salamandrids, the estimated rate of evolution given the two different calibrations was 0.091%/myr (primary calibration) and 0.197%/myr (additional calibrations; see [7]). These rates are higher than those estimated for our other nuclear loci. For our analyses, we used the *Plethodon* calibration.

References

1. Yang Z. PAML: a program package for phylogenetic analysis by maximum likelihood. Comput Appl Biosci. 1997;13: 555–556.

2. Thorne JL, Kishino H, Painter IS. Estimating the rate of evolution of the rate of molecular evolution. Mol Biol Evol. 1998;15: 1647–1657.

3. Kishino H, Thorne JL, Bruno WJ. Performance of a divergence time estimation method under a probabilistic model of rate evolution. Mol Biol Evol. 2001;18: 352–361.

4. Thorne JL, Kishino H. Divergence time and evolutionary rate estimation with multilocus data. Syst Biol. 2002;51: 689–702. doi:10.1080/10635150290102456

5. Zhang P, Papenfuss TJ, Wake MH, Qu L, Wake DB. Phylogeny and biogeography of the family Salamandridae (Amphibia: Caudata) inferred from complete mitochondrial genomes. Mol Phylogenet Evol. 2008;49: 586–597. doi:10.1016/j.ympev.2008.08.020

6. Sequeira F, Ferrand N, Harris DJ. Assessing the phylogenetic signal of the nuclear b-Fibrinogen intron 7 in salamandrids (Amphibia: Salamandridae). Amphibia-Reptilia. Springer; 2006;27: 409–418.

7. Zhang P, Chen Y-Q, Zhou H, Liu Y-F, Wang X-L, Papenfuss TJ, et al. Phylogeny, evolution, and biogeography of Asiatic Salamanders (Hynobiidae). Proceedings of the National Academy of Sciences of the United States of America. 2006;103: 7360–7365. doi:10.1073/pnas.0602325103

Table B. Species and indentication numbers of taxa and loci used in the multidivtime analysis. GenBank numbers are provided.

Species	Cyt b	ND2	tRNAs	MLC2A	GAPD	RAG1	ILF3	BFI
albagula	DQ994905.1	DQ018689.1	DQ018689	JN798307.1	JN798216.1	DQ995008	JN798264.1	–
amplus	DQ994912.1	AY874880.1	AY874880.1	JN798308.1	JN798217.1	DQ995010	JN798265.1	–
angusticla	DQ994913.1	DQ018676.1	DQ018676.1	–	JN798218.1	DQ995011	–	–
caddoensis	DQ994916.1	AY874875.1	AY874875.1	JN798311.1	JN798221.1	DQ995013	JN798268.1	–
chattahooc	DQ994919.1	–	–	JN798312.1	JN798222.1	DQ995014	JN798269.1	–
cheoah	DQ994921.1	–	–	JN798313.1	JN798223.1	DQ995015	JN798270.1	–
chlorobryo	DQ994923.1	–	–	JN798314.1	JN798224.1	–	JN798271.1	–
cinereus	AY378030	AY874875.1	AY874875.1	JN798317.1	JN798226.1	DQ995017	–	–
cylindrace	DQ994928.1	–	–	JN798318.1	JN798229.1	DQ995022	JN798272.1	–
dorsalis	AY378077.1	DQ018678.1	DQ018678.2	JN798319.1	–	DQ995023	JN798273.1	–
electromor	DQ994932.1	DQ018665.1	DQ018665.2	JN798320.1	JN798230.1	DQ995025	–	–
elongatus	AY691746.1	–	–	–	–	–	–	–
fourchensi	DQ994933.1	AY874876.1	AY874876.2	JN798321.1	JN798232.1	DQ995026	JN798274.1	–
glutinosus	–	–	–	–	–	–	–	KU841065
grobmani	DQ994941.1	DQ018695.1	DQ018695.2	JN798325.1	JN798236.1	DQ995028	JN798277.1	–
hoffmani	AY378048.1	DQ018666.1	DQ018666.2	JN798327.1	JN798238.1	DQ995029	–	–
hubrichti	AY378058.1	DQ018667.1	DQ018667.2	JN798328.1	JN798239.1	DQ995030	–	–
idahoensis	DQ994945.1	–	–	JN798329.1	–	DQ995031	–	–
jordani	DQ994947.1	AY874887.1	AY874887.2	JN798330.1	–	DQ995032	JN798278.1	–
kentucki	DQ994948.1	KU841032	KU841032	JN798331.1	JN798240.1	DQ995033	JN798279.1	KU841122
kiamichi	DQ994950.1	DQ018696.1	DQ018696.2	JN798332.1	JN798241.1	DQ995034	JN798280.1	–
kisatchie	DQ994951.1	DQ018698.1	DQ018698.2	JN798333.1	JN798242.1	DQ995035	JN798281.1	–
meridianus	DQ994954.1	AY874898.1	AY874898.2	JN798335.1	JN798244.1	DQ995038	JN798283.1	–
metcalfi	DQ994956.1	AY874903.1	AY874903.2	–	JN798245.1	DQ995040	JN798284.1	–
mississipp	DQ994958.1	DQ018700.1	DQ018700.2	–	JN798246.1	–	JN798285.1	–
montanus	DQ994961.1	AY874960.1	AY874960.2	JN798336.1	JN798247.1	DQ995042	JN798286.1	–
nettingi	DQ994964.1	–	–	–	JN798248.1	DQ995045	–	–

Species	Cyt b	ND2	tRNAs	MLC2A	GAPD	RAG1	ILF3	BFI
ocmulgee	DQ994967.1	DQ018702.1	DQ018702.2	JN798338.1	JN798250.1	DQ995048	JN798288.1	–
ouachitae	Y691747.1	AY874877.1	AY874877.2	–	JN798251.1	–	JN798289.1	–
petraeus	DQ994973.1	AY874878.1	AY874878.2	JN798339.1	JN798252.1	DQ995049	JN798290.1	–
punctatus	AY378078.1	DQ018685.1	DQ018685.2	–	JN798253.1	DQ995050	JN798291.1	–
richmondi	AY378070.1	DQ018670.1	DQ018670.2	JN798340.1	JN798254.1	DQ995051	–	–
savannah	DQ994978.1	–	–	JN798341.1	JN798255.1	DQ995055	JN798292.1	–
sequoyah	DQ994979.1	DQ018705.1	–	JN798342.1	–	DQ995056	JN798293.1	–
serratus	AY378073.1	DQ018671.1	DQ018671.2	JN798343.1	JN798256.1	DQ995057	–	–
shenandoah	AY378043.1	DQ018674.1	DQ018674.2	JN798345.1	JN798257.1	DQ995062	–	–
teyahalee	DQ994990.1	–	–	JN798347.1	–	DQ995068	JN798295.1	–
vandykei	AY691759.1	–	–	–	JN798260.1	–	–	–
variolatus	DQ994991.1	–	–	JN798348.1	–	DQ995069	JN798296.1	–
vehiculum	AY691760.1	DQ018661.1	DQ018661.2	JN798349.1	–	–	–	–
ventralis	DQ994993.1	DQ018680.1	DQ018680.2	JN798350.1	JN798261.1	DQ995071	JN798297.1	–
virginia	AY378049.1	DQ018675.1	DQ018675.2	JN798351.1	–	DQ995072	JN798298.1	–
websteri	AY378076.1	–	–	JN798352.1	–	DQ995073	JN798299.1	–
wehrlei	AY378079.1	DQ018686.1	DQ018686.2	JN798353.1	–	DQ995075	JN798300.1	KU863657
welleri	AY691761.1	–	–	–	JN798262.1	–	JN798301.1	–
yonahlosse	AY378075.1	AY874879.1	AY874879.2	JN798354.1	JN798263.1	DQ995078	JN798302.1	–

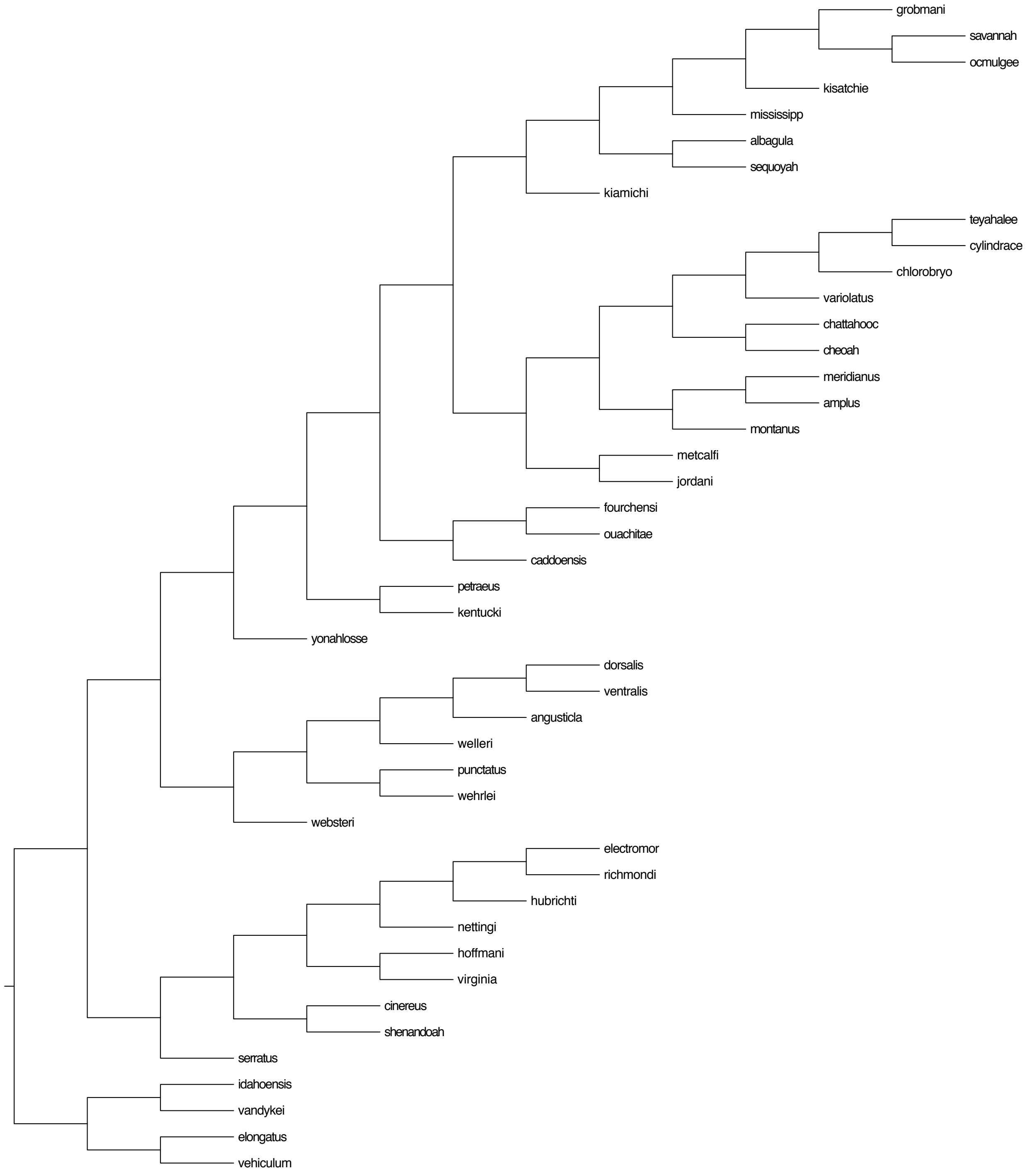

Figure A. Phylogeny of *Plethodon* used in the Multidivtime analyses. See text for details.

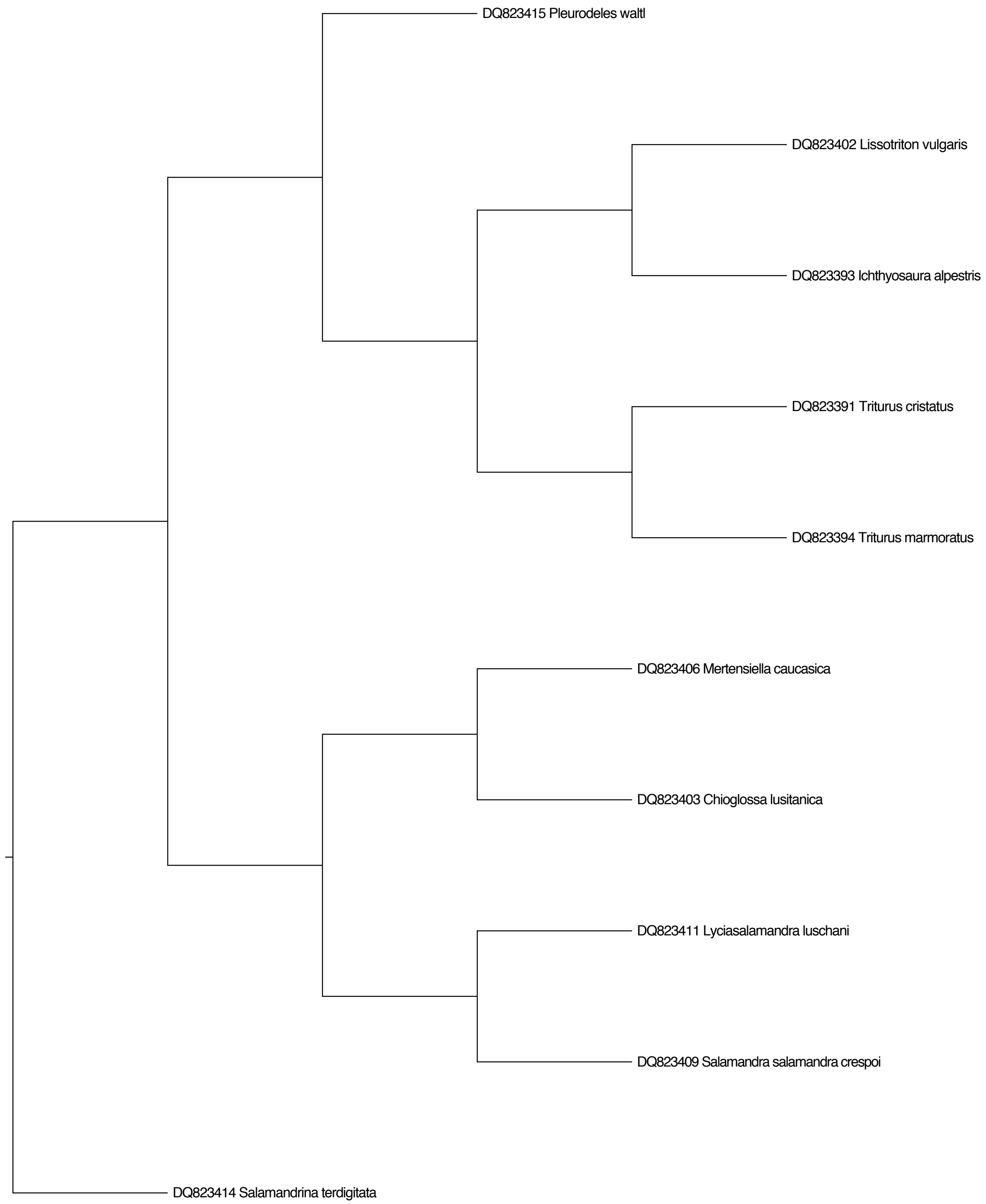

Figure B. Phylogeny of Salamandridae **used in the Multidivtime analyses**. See text for details.
