# Supplementary material for: Multilocus Phylogeography and Species Delimitation in the Cumberland Plateau Salamander, Plethodon kentucki: Incongruence among Data Sets and Methods
Source: PLoS One. 2016 Mar 14;11(3):e0150022. doi: 10.1371/journal.pone.0150022 (PMC4790894; doi:10.1371/journal.pone.0150022)
Supplement: S3 Appendix — Figures C-G. (PDF) [file pone.0150022.s003.pdf]

Appendix S3

Gene trees for nuclear loci

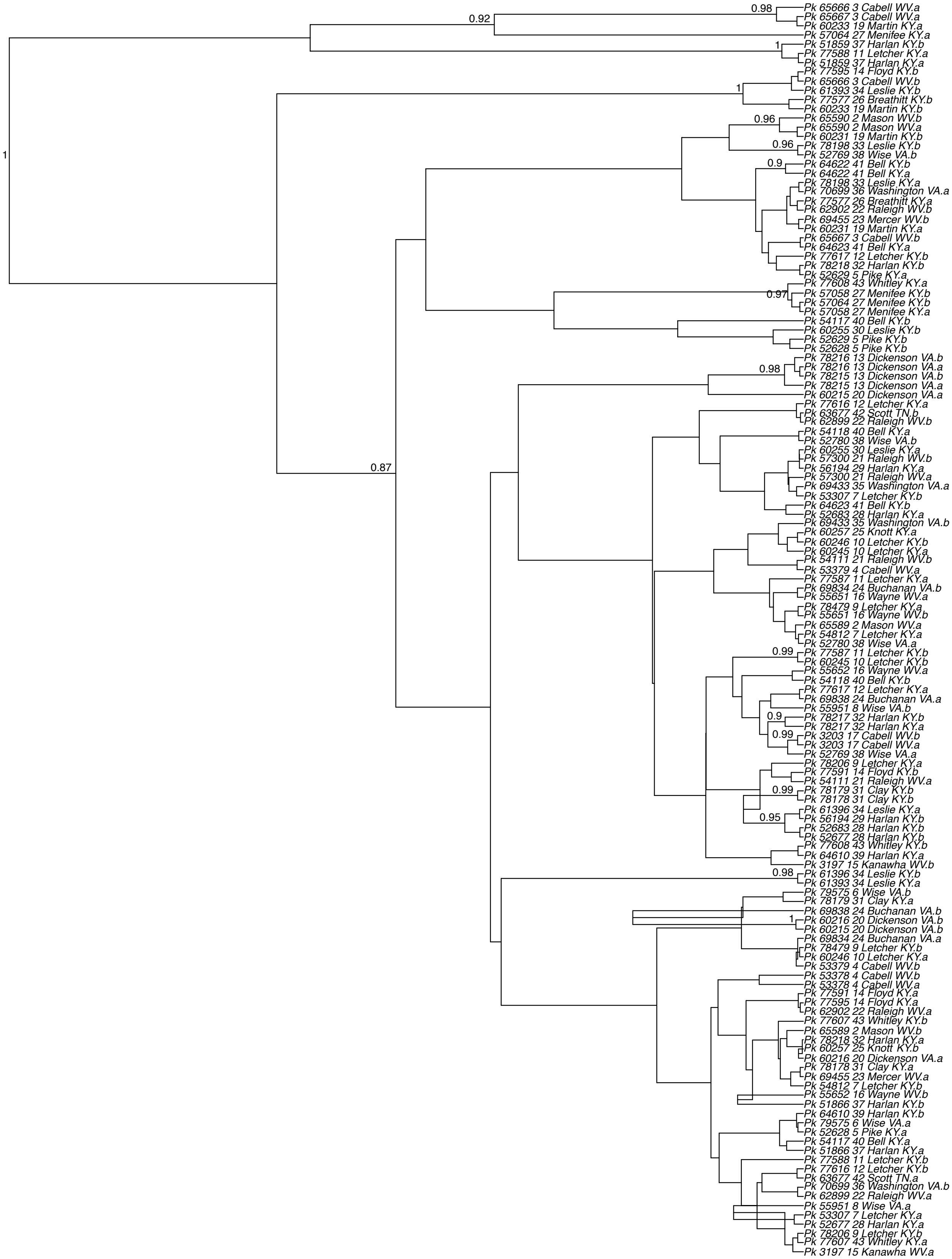

Figure C. Gene tree of BFI. Specimen numbers correspond with the S1 Appendix, and population numbers correspond with Figure 1 in the text.

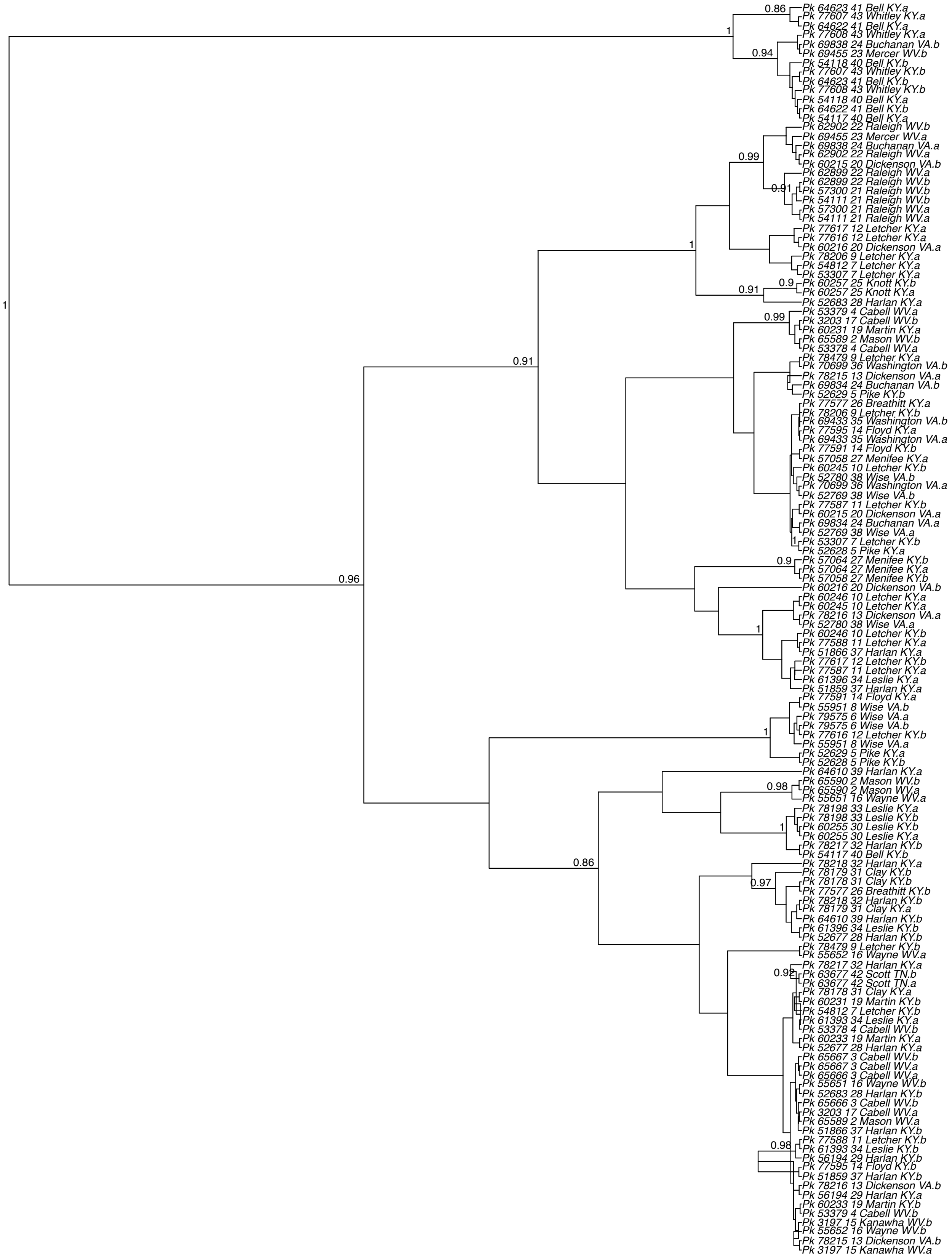

Figure D. Gene tree of GAPD. Specimen numbers correspond with the S1 Appendix, and population numbers correspond with Figure 1 in the text.

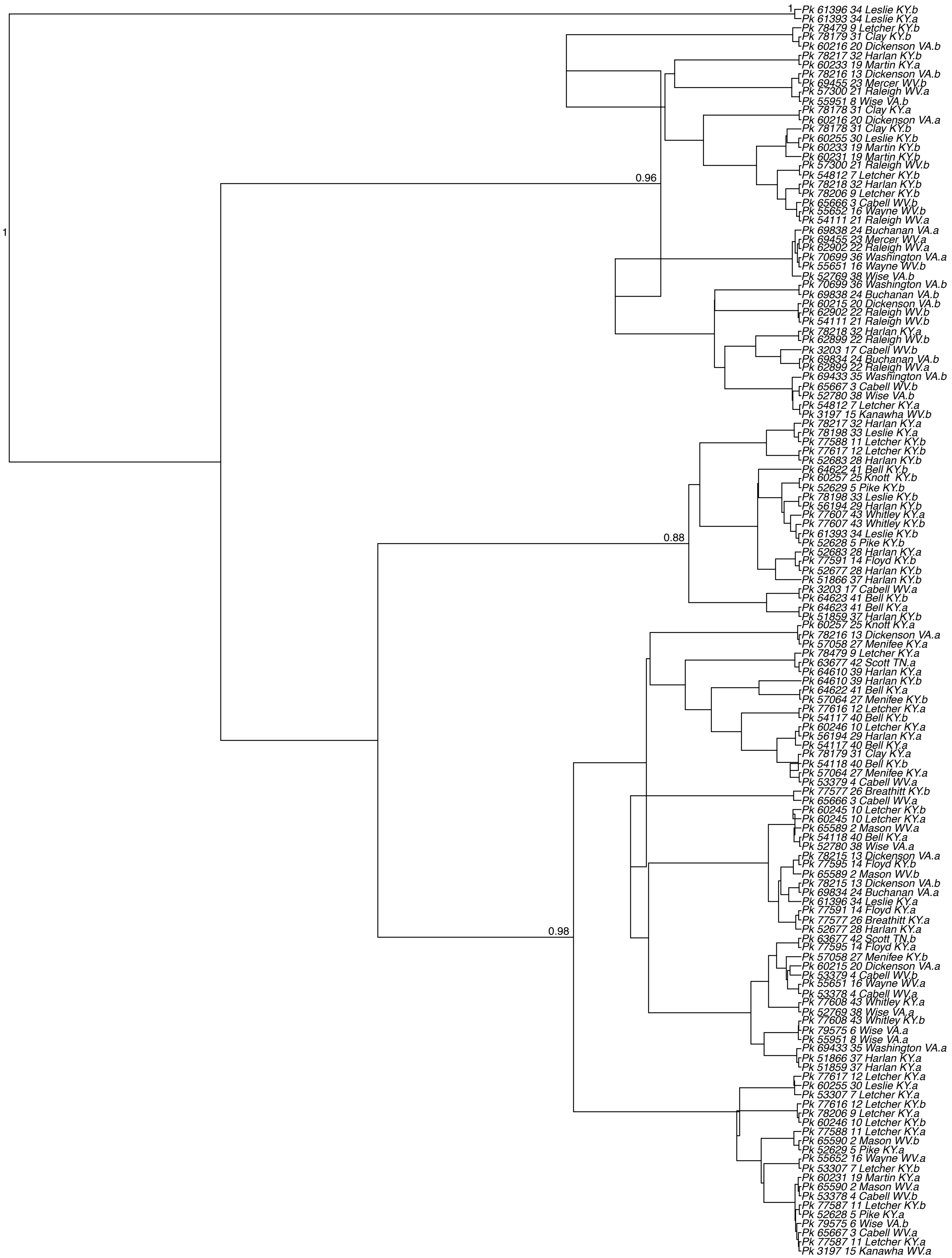

Figure E. Gene tree of ILF3. Specimen numbers correspond with the S1 Appendix, and population numbers correspond with Figure 1 in the text.

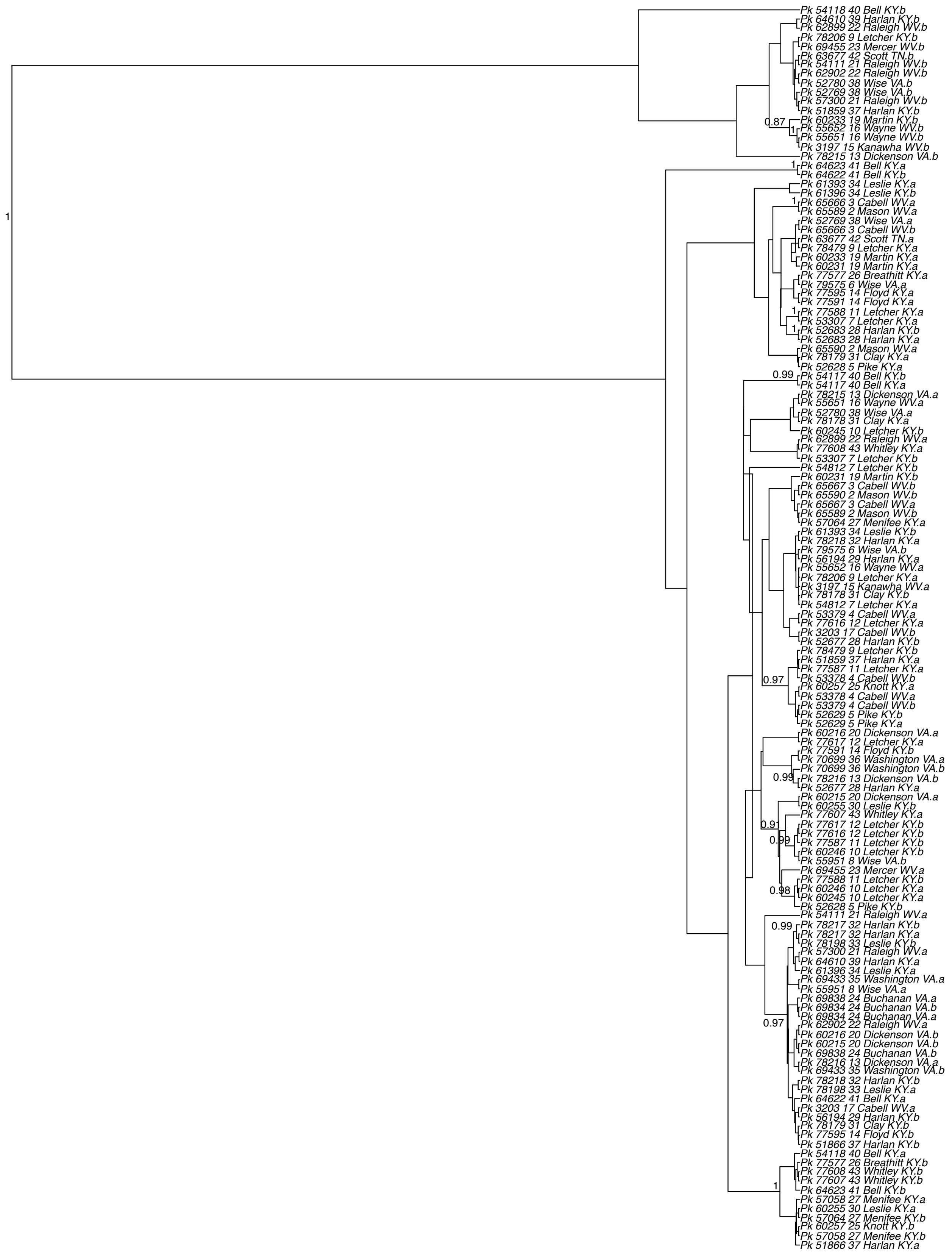

Figure F. Gene tree of MLC2A. Specimen numbers correspond with the S1 Appendix, and population numbers correspond with Figure 1 in the text.

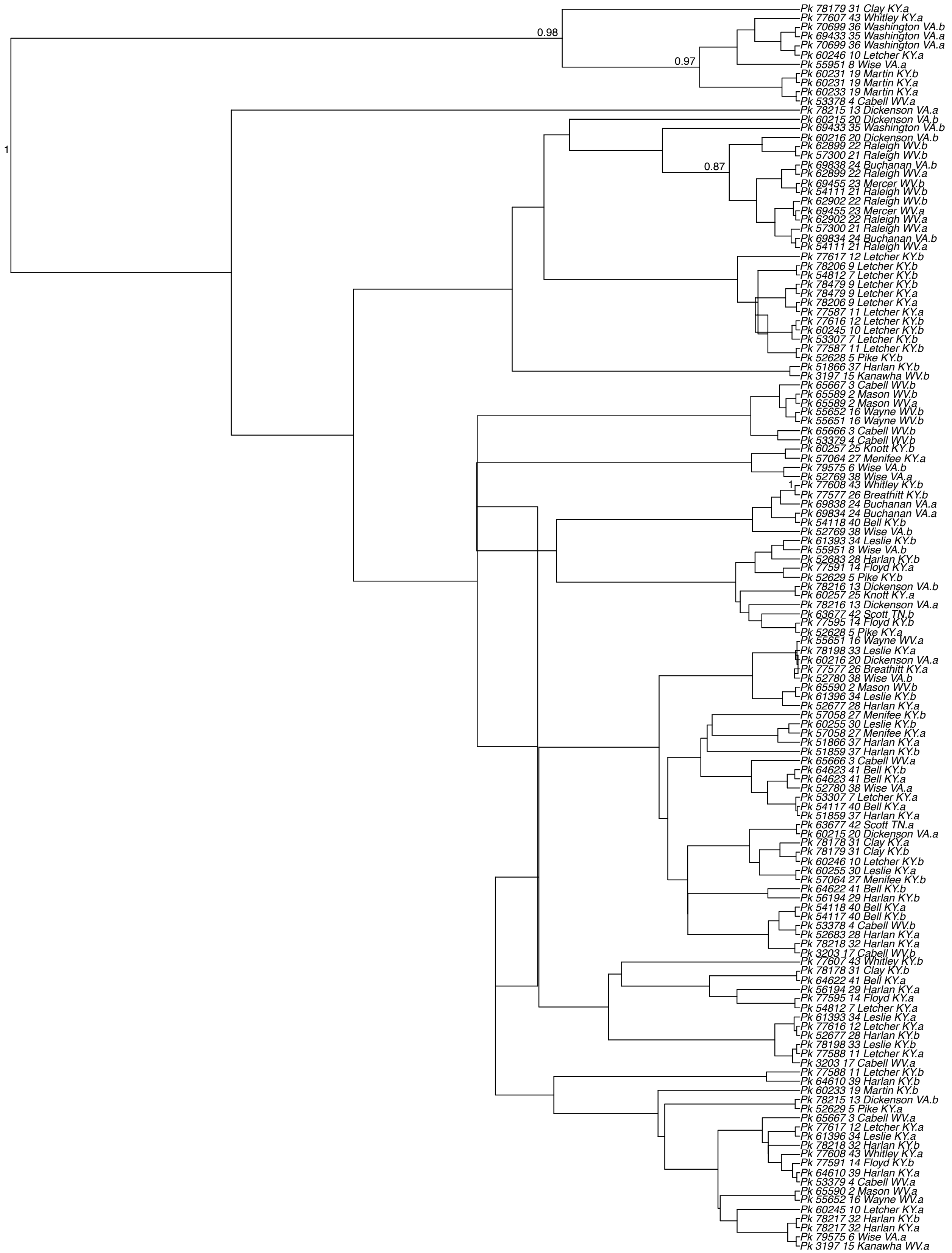

Figure G. Gene tree of RAG-1. Specimen numbers correspond with the S1 Appendix, and population numbers correspond with Figure 1 in the text.
