# Supplementary material for: Multilocus Phylogeography and Species Delimitation in the Cumberland Plateau Salamander, Plethodon kentucki: Incongruence among Data Sets and Methods
Source: PLoS One. 2016 Mar 14;11(3):e0150022. doi: 10.1371/journal.pone.0150022 (PMC4790894; doi:10.1371/journal.pone.0150022)
Supplement: S4 Appendix — Figures H-I. (PDF) [file pone.0150022.s004.pdf]

Appendix S4

Concatenated analyses using RAxML, including a phylogeny of the nuclear loci, and a phylogeny with both nuclear and mtDNA loci.

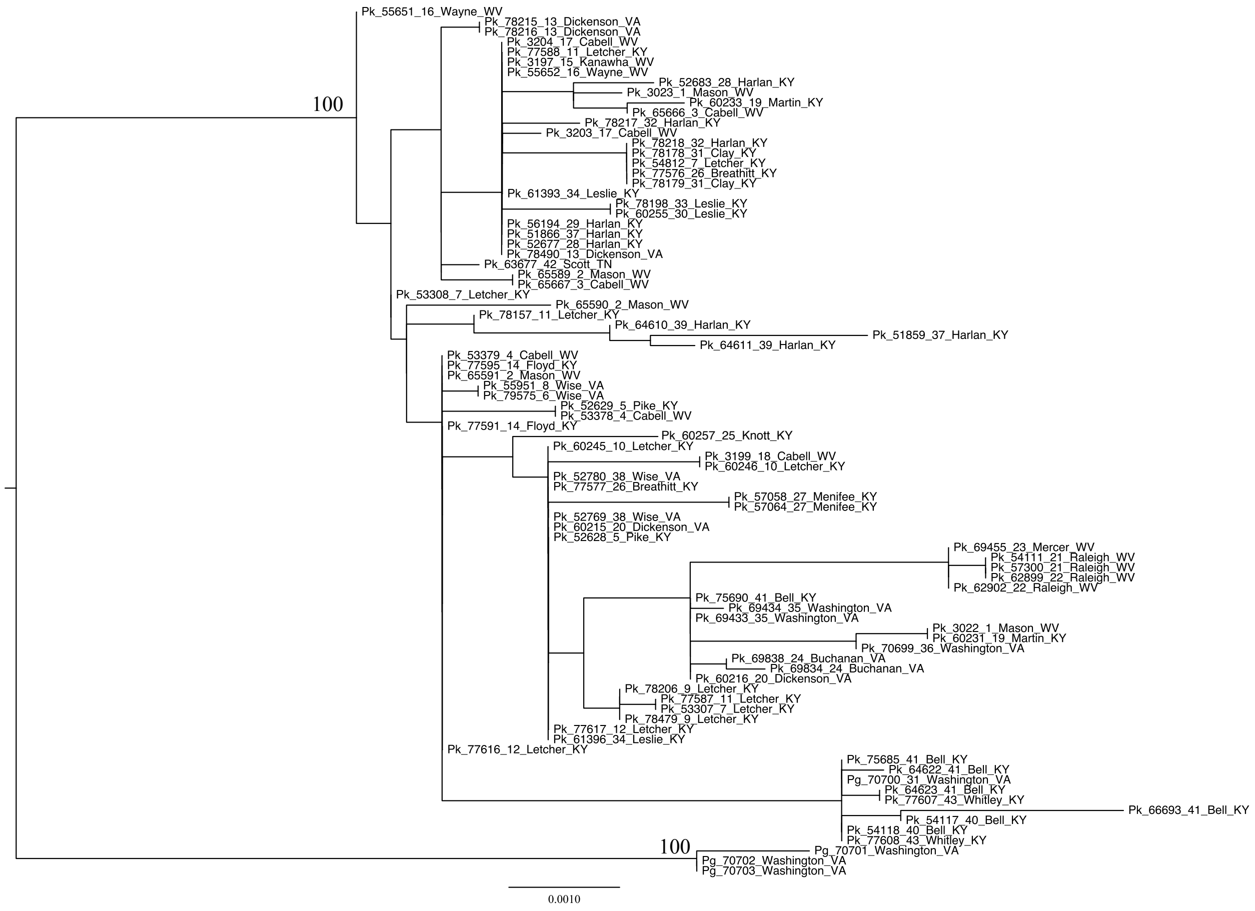

Figure H. Maximum likelihood analysis of the concatenated nuclear DNA data. MtDNA data not included. Analysis done in RAxML.

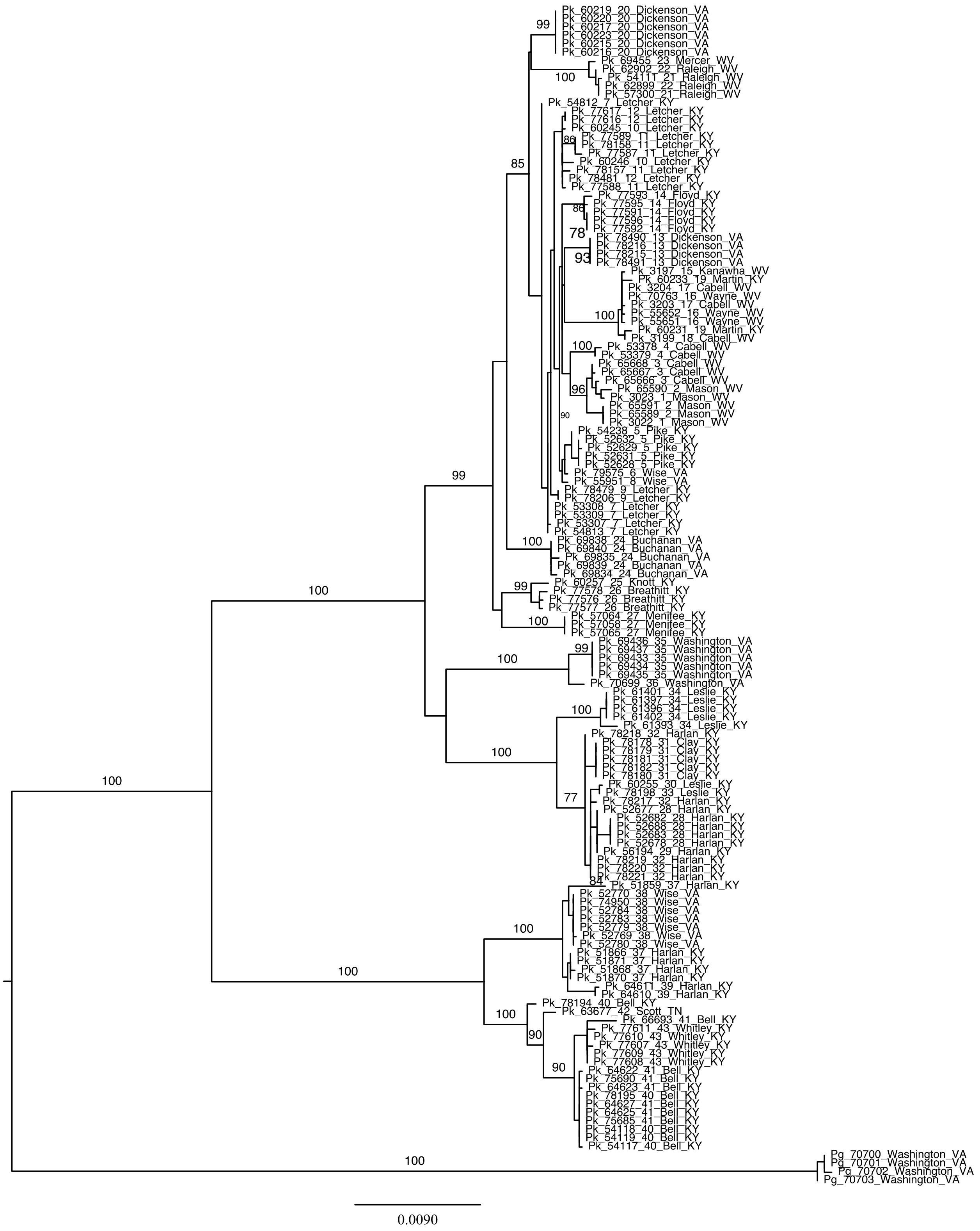

Figure I. Maximum likelihood analysis of the concatenated nuclear and mtDNA data. Analysis done in RAxML.
