# Supplementary material for: Multilocus Phylogeography and Species Delimitation in the Cumberland Plateau Salamander, Plethodon kentucki: Incongruence among Data Sets and Methods
Source: PLoS One. 2016 Mar 14;11(3):e0150022. doi: 10.1371/journal.pone.0150022 (PMC4790894; doi:10.1371/journal.pone.0150022)
Supplement: S5 Appendix — Results, Tables C-H, Figures J-K. (PDF) [file pone.0150022.s005.pdf]

Appendix S5: Brownie Results

We analyzed numerous data sets using Brownie, as described in the Materials and Methods in the main text. When the nuclear data alone were analyzed, four replicate data sets delimited non-overlapping species, with the number of delimited species in data sets 1-3 ranging from 5-23 (Tables C-E). In data set 4, 2333 unique species trees were recovered, with 2007 separate delimited species. A majority rule consensus tree recovered one species including three individuals from populations 14 and 28 (Fig. J), but the other individuals were all placed into a basal polytomy. In all analyses, the delimited species did not include all the individuals from a population, and did not form geographically cohesive groups.

In a second set of analyses, we added mtDNA data to the nuclear data sets. For data set 1, all 500 runs recovered *P. kentucki* as a single species. In data set 2, two species were recovered, one including single individuals from populations 6, 9, and 17, and the other including all the other samples (Table F). The first species includes individuals from widely spaced populations, and does not include all the individuals from the included populations. In data set 3, 12618 trees were recovered from the 500 runs, including 8556 unique species tree topologies and 5018 delimited species. A majority rule consensus tree delimited three species, each with two individuals, while all other individuals formed a basal polytomy (Fig. K). Again, of the three delimited species, none formed a geographically cohesive group or included all of the individuals from their respective populations. In data set 4, two species were recovered in all 500 runs, one including a single individual from each of populations 7 and 19, and the other including all the remaining samples (Table G). The former does not form a geographically cohesive

group, or include all the individuals from the included populations. In summary, when we combined mtDNA and nuclear DNA, the delimited species varied widely among analyses. Only the run that delimited *P. kentucki* as a single species recovered a species that is geographically cohesive and does not split individuals from a single population into separate species.

Finally, we conducted an analysis of the diploid nuclear data, with both alleles from each individual included. This doubles the sample size, but does not account for the hierarchical structure of genetic material (i.e., that there are two alleles/individual). Niemiller et al. [1] found that including a phased nuclear data set increased the number of delimited species, as did increasing the number of individuals and loci sampled. In general, they recommended that a single allele per individual be used, but that analyses on phased data are valuable to determine if heterozygous individuals are split into separate species. Our analysis of the diploid data set recovered 24 unique species trees and 36 species (Table H). In every delimited species, alleles from a single individual were separated into separate species.

In sum, Brownie did not reliably delimit species. Vast differences were recovered when different data sets were used. The delimited species did not form contiguous geographic groups, and typically did not include all the individuals from single populations. This was true whether or not mtDNA was included. Thus, we did not further test the validity of these species.

Reference

1. Niemiller ML, Near TJ, Fitzpatrick BM. Delimiting species using multilocus data: diagnosing cryptic diversity in the southern cavefish, *Typhlichthys subterraneus* (Teleostei: Amblyopsidae). Evolution. 2011;66: 846–866. doi:10.1111/j.1558-5646.2011.01480.x

Table C. Species delimінted by Brownie using data set 1. The results of 500 independent runs are shown here. Grey boxes indicate inclusion in a partition. 1811 trees were recovered because many analyses recovered 2-4 trees of equal length. The alleles in this set of runs and the set illustrated in Table S2 do not overlap for heterozygous individuals.

	**Number**	**1431**	**1376**	**1695**	**815**	**551**	**440**	**112**	**108**	**59**	**3**	**4**
	**Freq**	**0.790**	**0.760**	**0.936**	**0.450**	**0.304**	**0.243**	**0.062**	**0.060**	**0.033**	**0.002**	**0.002**
**Specimen**	**Population**											
65589	2											
65590	2											
65666	3											
65667	3											
53378	4											
53379	4											
52628	5											
52629	5											
79575	6											
53307	7											
54812	7											
55951	8											
78206	9											
78479	9											
60245	10											
60246	10											
77587	11											
77588	11											
77616	12											
77617	12											
78215	13											
78216	13											
77591	14											
77595	14											
3197	15											
55651	16											
55652	16											
3203	17											
60231	19											
60233	19											
60215	20											
60216	20											
54111	21											
57300	21											
62899	22											
62902	22											
69455	23											
69834	24											
69838	24											
60257	25											
77577	26											
57058	27											
57064	27											
52677	28											
52683	28											
56194	29											
60255	30											
78178	31											
78179	31											
78217	32											
78218	32											
78198	33											
61393	34											
61396	34											
69433	35											
70699	36											
51859	37											
51866	37											
52769	38											
52780	38											
64610	39											
54117	40											
54118	40											
64622	41											
64623	41											
63677	42											
77607	43											
77608	43											

Table D. Species deliminted by Brownie using data set 2. The results of 500 independent runs are shown here. Grey boxes indicate inclusion in a partition. 500 trees were recovered (1/run). The alleles in this set of runs and the set illustrated in Table S do not overlap for heterozygous individuals.

	**Number**	**500**	**499**	**1**	**1**	**1**
	**Freq**	**1.000**	**0.998**	**0.002**	**0.002**	**0.002**
**Specimen**	**Population**					
65589	2		X	X	X	
65590	2		X	X	X	
65666	3		X	X	X	
65667	3		X	X	X	
53378	4		X	X	X	
53379	4		X	X	X	
52628	5		X	X	X	
52629	5		X	X	X	
79575	6		X	X	X	
53307	7		X	X	X	
54812	7		X	X	X	
55951	8		X	X	X	
78206	9		X	X	X	
78479	9	X			X	
60245	10		X	X	X	
60246	10		X	X	X	
77587	11		X	X	X	
77588	11		X	X	X	
77616	12		X	X	X	
77617	12		X	X	X	
78215	13		X	X	X	
78216	13		X	X	X	
77591	14		X	X	X	
77595	14		X	X	X	
3197	15		X	X	X	
55651	16		X	X	X	
55652	16		X	X	X	
3203	17	X			X	
60231	19		X	X	X	
60233	19		X	X	X	
60215	20		X	X	X	
60216	20		X	X	X	
54111	21		X	X	X	
57300	21		X	X	X	
62899	22		X	X	X	
62902	22		X	X	X	
69455	23		X	X	X	
69834	24		X	X	X	
69838	24		X	X	X	
60257	25		X	X	X	
77577	26		X	X	X	
57058	27		X			X
57064	27		X			X
52677	28		X	X	X	
52683	28		X	X	X	
56194	29		X	X	X	
60255	30		X	X	X	
78178	31		X	X	X	
78179	31		X	X	X	
78217	32		X	X	X	
78218	32		X	X	X	
78198	33		X	X	X	
61393	34		X	X	X	
61396	34		X	X	X	
69433	35		X	X	X	
70699	36		X	X	X	
51859	37		X	X	X	
51866	37		X	X	X	
52769	38		X	X	X	
52780	38		X	X	X	
64610	39		X	X	X	
54117	40		X	X	X	
54118	40		X	X	X	
64622	41		X	X	X	
64623	41		X	X	X	
63677	42		X	X	X	
77607	43		X	X	X	
77608	43		X	X	X	

Table E. Species delimintted by Brownie using DS3. The results of 500 independent runs are shown here. Grey boxes indicate inclusion in a partition. 503 trees were recovered because some analyes recovered two trees of equal length.

	**Number**	**498**	**495**	**7**	**5**	**3**	**2**	**2**	**1**	**1**	**1**	**1**	**1**	**1**	**1**	**1**	**1**	**1**	**1**	**1**	**1**	**1**	**1**	**1**
	**Freq**	**0.990**	**0.984**	**0.014**	**0.010**	**0.006**	**0.004**	**0.004**	**0.002**	**0.002**	**0.002**	**0.002**	**0.002**	**0.002**	**0.002**	**0.002**	**0.002**	**0.002**	**0.002**	**0.002**	**0.002**	**0.002**	**0.002**	**0.002**
**Specimen**	**Population**																							
65589	2																							
65590	2																							
65666	3																							
65667	3																							
53378	4																							
53379	4																							
52628	5																							
52629	5																							
79575	6																							
53307	7																							
54812	7																							
55951	8																							
78206	9																							
78479	9																							
60245	10																							
60246	10																							
77587	11																							
77588	11																							
77616	12																							
77617	12																							
78215	13																							
78216	13																							
77591	14																							
77595	14																							
3197	15																							
55651	16																							
55652	16																							
3203	17																							
60231	19																							
60233	19																							
60215	20																							
60216	20																							
54111	21																							
57300	21																							
62899	22																							
62902	22																							
69455	23																							
69834	24																							
69838	24																							
60257	25																							
77577	26																							
57058	27																							
57064	27																							
52677	28																							
52683	28																							
56194	29																							
60255	30																							
78178	31																							
78179	31																							
78217	32																							
78218	32																							
78198	33																							
61393	34																							
61396	34																							
69433	35																							
70699	36																							
51859	37																							
51866	37																							
52769	38																							
52780	38																							
64610	39																							
54117	40																							
54118	40																							
64622	41																							
64623	41																							
63677	42																							
77607	43																							
77608	43																							

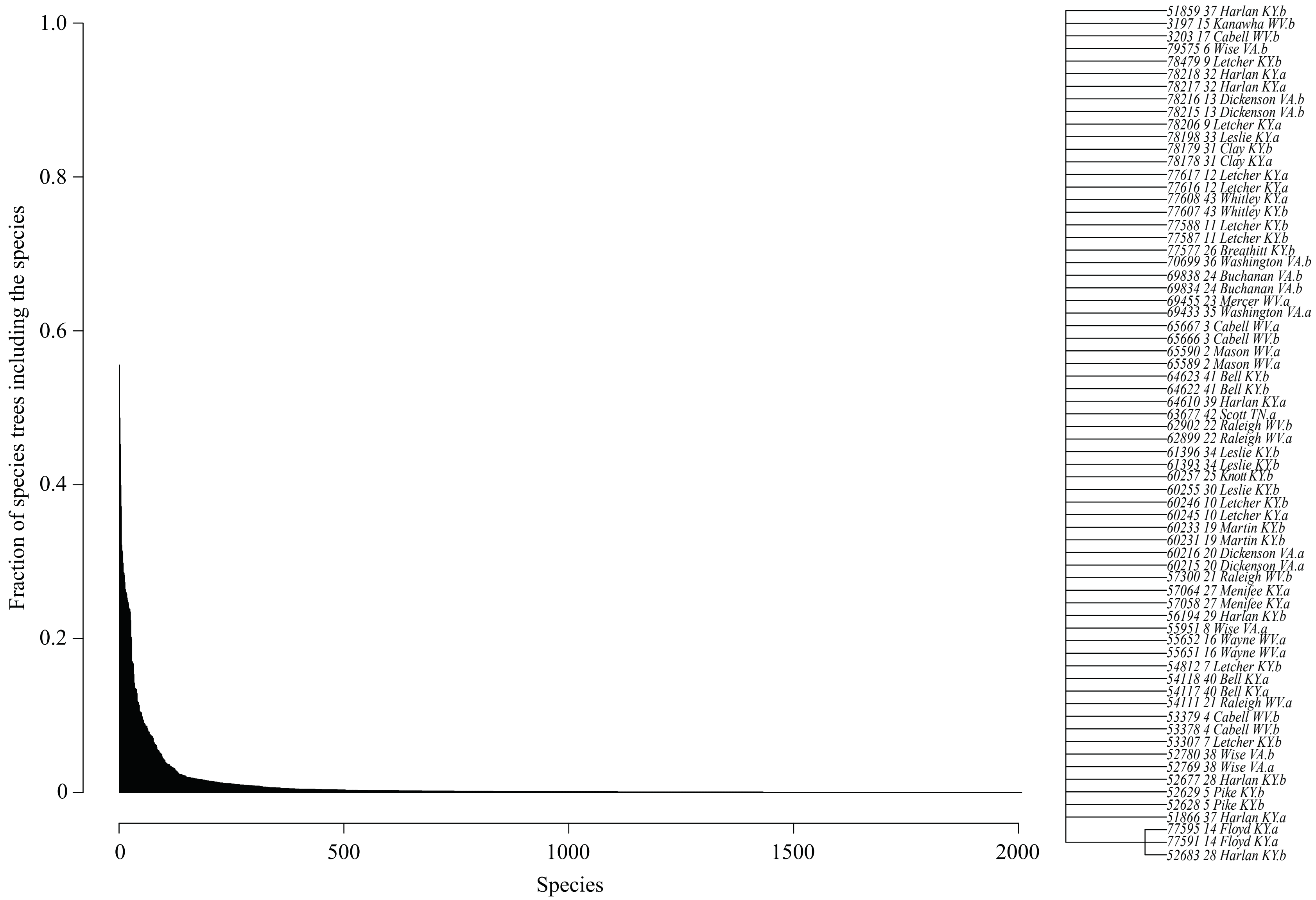

Fig. J. Summary of 500 replicate runs in Brownie with data set 4. 2333unque species tree topologies were recovered, including 2007delimited species. The graph shows that that most of these species were recovered in a small number of trees. A majority rule consensus tree is provided in the right portion of the figure, showing the one species that was recovered in >50% of trees.

Table F. Species delimintd by Brownie using data set 2 with mtDNA. The results of 500 independent runs are shown here. Grey boxes indicate inclusion in a delimited species. Two species were consistently delimited in this analysis

	**Number**	**500**	**500**
	**Freq**	**1.0**	**1.0**
**Specimen**	**Population**		[grey]
65589	2		[grey]
65590	2		[grey]
65666	3		[grey]
65667	3		[grey]
53378	4		[grey]
53379	4		[grey]
52628	5		[grey]
52629	5		[grey]
79575	6	[grey]	
53307	7		[grey]
54812	7		[grey]
55951	8		[grey]
78206	9		[grey]
78479	9	[grey]	
60245	10		[grey]
60246	10		[grey]
77587	11		[grey]
77588	11		[grey]
77616	12		[grey]
77617	12		[grey]
78215	13		[grey]
78216	13		[grey]
77591	14		[grey]
77595	14		[grey]
3197	15		[grey]
55651	16		[grey]
55652	16		[grey]
3203	17	[grey]	
60231	19		[grey]
60233	19		[grey]
60215	20		[grey]
60216	20		[grey]
54111	21		[grey]
57300	21		[grey]
62899	22		[grey]
62902	22		[grey]
69455	23		[grey]
69834	24		[grey]
69838	24		[grey]
60257	25		[grey]
77577	26		[grey]
57058	27		[grey]
57064	27		[grey]
52677	28		[grey]
52683	28		[grey]
56194	29		[grey]
60255	30		[grey]
78178	31		[grey]
78179	31		[grey]
78217	32		[grey]
78218	32		[grey]
78198	33		[grey]
61393	34		[grey]
61396	34		[grey]
69433	35		[grey]
70699	36		[grey]
51859	37		[grey]
51866	37		[grey]
52769	38		[grey]
52780	38		[grey]
64610	39		[grey]
54117	40		[grey]
54118	40		[grey]
64622	41		[grey]
64623	41		[grey]
63677	42		[grey]
77607	43		[grey]
77608	43		[grey]

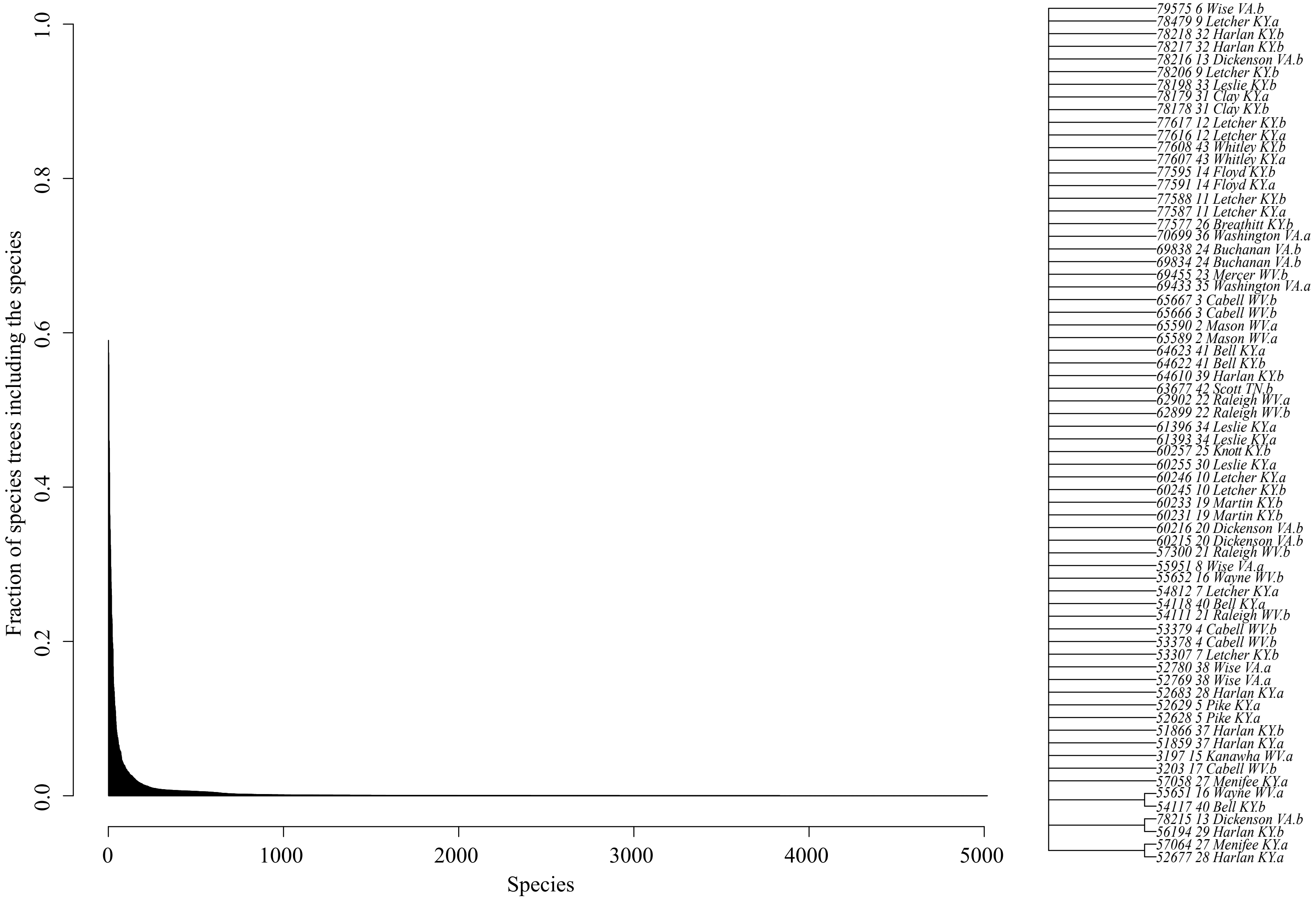

Fig. K. Summary of 500 replicate runs in Brownie, using data set 3 with mtDNA. 8556 unque species tree topologies were recovered, including 5018 delimited species. The graph shows that that most of these species were recovered in a single tree. A majority rule consensus tree is provided in the right portion of the figure, showing the three species that were recovered in >50% of trees.

Table G. Species delimINTED by Brownie using data set 4 with mtDNA. The results of 500 independent runs are shown here. Grey boxes indicate inclusion in a delimited species. Two species were consistently delimited in this analysis

	**Number**	**500**	**500**
	**Freq**	**1.0**	**1.0**
**Specimen**	**Population**		
65589	2		■
65590	2		■
65666	3		■
65667	3		■
53378	4		■
53379	4		■
52628	5		■
52629	5		■
79575	6		■
53307	7		■
54812	7	■	
55951	8		■
78206	9		■
78479	9		■
60245	10		■
60246	10		■
77587	11		■
77588	11		■
77616	12		■
77617	12		■
78215	13		■
78216	13		■
77591	14		■
77595	14		■
3197	15		■
55651	16		■
55652	16		■
3203	17		■
60231	19	■	
60233	19		■
60215	20		■
60216	20		■
54111	21		■
57300	21		■
62899	22		■
62902	22		■
69455	23		■
69834	24		■
69838	24		■
60257	25		■
77577	26		■
57058	27		■
57064	27		■
52677	28		■
52683	28		■
56194	29		■
60255	30		■
78178	31		■
78179	31		■
78217	32		■
78218	32		■
78198	33		■
61393	34		■
61396	34		■
69433	35		■
70699	36		■
51859	37		■
51866	37		■
52769	38		■
52780	38		■
64610	39		■
54117	40		■
54118	40		■
64622	41		■
64623	41		■
63677	42		■
77607	43		■
77608	43		■

Table H. Species delimited by Brownie using the diploid data. The results of 500 independent runs are shown here. Grey boxes indicate inclusion in a delimited species. 24 unique species trees were recoveredin the analyses.

Number	1	1	1	1	36	12	35	10	31	1164	10	11	6	9	6	383	1132	377	1462	708	1	1	1	1	1	1	1	336	112	432	336	112	208	1	1	1
Frequency	0.0005	0.0005	0.0005	0.0005	0.0186	0.0062	0.0181	0.0052	0.0160	0.6022	0.0052	0.0057	0.0031	0.0047	0.0031	0.1981	0.5856	0.1950	0.7563	0.3663	0.0005	0.0005	0.0005	0.0005	0.0005	0.0005	0.0005	0.1738	0.0579	0.2235	0.1738	0.0579	0.1076	0.0005	0.0005	0.0005

Specimen	Population
65589	2
65589	2
65590	2
65590	2
65666	3
65666	3
65667	3
65667	3
53378	4
53378	4
53379	4
53379	4
52628	5
52628	5
52629	5
52629	5
79575	6
79575	6
53307	7
53307	7
54812	7
54812	7
55951	8
55951	8
78206	9
78206	9
78479	9
78479	9
60245	10
60245	10
60246	10
60246	10
77587	11
77587	11
77588	11
77588	11
77616	12
77616	12
77617	12
77617	12
78215	13
78215	13
78216	13
78216	13
77591	14
77591	14
77595	14
77595	14
3197	15
3197	15
55651	16
55651	16
55652	16
55652	16
3203	17
3203	17
60231	19
60231	19
60233	19
60233	19
60215	20
60215	20
60216	20
60216	20
54111	21
54111	21
57300	21
57300	21
62899	22
62899	22
62902	22
62902	22
69455	23
69455	23
69834	24
69834	24
69838	24
69838	24
60257	25
60257	25
77577	26
77577	26
57058	27
57058	27
57064	27
57064	27
52677	28
52677	28
52683	28
52683	28
56194	29
56194	29
60255	30
60255	30
78178	31
78178	31
78179	31
78179	31
78217	32
78217	32
78218	32
78218	32
78198	33
78198	33
61393	34
61393	34
61396	34
61396	34
69433	35
69433	35
70699	36
70699	36
51859	37
51859	37
51866	37
51866	37
52769	38
52769	38
52780	38
52780	38
64610	39
64610	39
54117	40
54117	40
54118	40
54118	40
64622	41
64622	41
64623	41
64623	41
63677	42
63677	42
77607	43
77607	43
77608	43
77608	43
