# Supplementary material for: Multilocus Phylogeography and Species Delimitation in the Cumberland Plateau Salamander, Plethodon kentucki: Incongruence among Data Sets and Methods
Source: PLoS One. 2016 Mar 14;11(3):e0150022. doi: 10.1371/journal.pone.0150022 (PMC4790894; doi:10.1371/journal.pone.0150022)
Supplement: S6 Appendix — Table I. (PDF) [file pone.0150022.s006.pdf]

Appendix S6

Genealogical sorting index results

Table I. Genealogical sorting index results for putative species deliminted using bGMYC and Geneland. Data sets are described in the text. *P* values are based on 10,000 permutations, and are denoted with asterisks. *egsi* = ensamble gsi, for nuclear loci only. MtDNA is only reported for the Geneland analyses.

Delimitation	Data set	OTU	BFI	GAPD	ILF3	MLC2A	RAG1	*egsi*	mtDNA
bGMYC: 2 species	Data set 1	A	0.07	**0.45****	**0.29***	0.07	0.14	**0.20****	
		B	0.15	0.23	**0.29***	0.17	0.18	**0.20***	
	Data set 2	A	0.07	**0.37****	0.14	0.07	0.00	**0.13***	
		B	0.23	0.18	**0.27***	0.24	0.26	**0.24****	
	Data set 3	A	0.00	0.21	0.07	0.07	0.21	0.11	
		B	0.11	0.20	**0.29***	0.13	0.21	0.19	
	Data set 4	A	0.14	**0.29***	0.21	0.00	0.14	**0.16****	
		B	0.17	0.24	0.21	0.21	0.23	**0.21***	
	Diploid	A	0.14	**0.33****	0.11	0.14	0.00	**0.14****	
		B	0.23	**0.26***	0.23	0.21	0.20	**0.23****	
bGMYC: 17 species	Data set 1	A	0.14	0.24	0.16	0.21	0.19	**0.19***	
		B	0.09	0.19	0.14	0.11	0.14	0.13	
		C	0.10	0.16	0.19	0.10	0.15	0.14	
		D	0.15	**0.36****	0.25	0.14	0.13	**0.21***	
		E	0.12	**0.32***	0.16	0.13	0.20	0.19	
		F	0.09	0.49	0.05	0.13	0.05	0.16	
		G	0.11	**0.79****	**0.36****	0.16	**1.00****	**0.48****	
		H	0.20	**0.34****	0.18	0.17	0.11	**0.20***	
		I	0.15	0.13	0.10	0.11	0.10	0.12	
		J	0.09	0.07	0.06	0.24	0.10	0.11	
		K	0.06	0.09	0.10	0.05	0.08	0.07	
		L	0.11	0.05	0.08	0.09	0.09	0.08	
		M	0.05	0.24	0.06	0.11	0.49	0.19	
		N	0.06	0.06	0.06	**1.00***	**1.00***	**0.44***	
		O	0.49	0.06	0.11	1.00	0.09	**0.35***	
		Q	**1.00***	0.11	0.10	0.07	0.10	0.28	
	Data set 2	A	0.10	0.16	0.08	0.21	0.24	0.16	
		B	0.12	0.15	0.22	0.22	0.19	0.18	
		C	0.16	0.11	0.16	0.12	0.19	0.15	
		D	0.14	0.26	0.22	**0.26***	**0.32***	**0.24****	
		E	0.20	**0.30***	0.21	**0.28***	0.21	**0.24****	
		F	0.19	0.11	0.08	0.32	0.07	0.15	
		G	0.19	**0.36***	0.20	**0.36***	**0.54****	**0.33****	
		H	0.17	**0.31***	**0.29***	0.25	0.22	**0.25****	
		I	1.00	1.00	0.11	0.49	0.15	**0.55****	
		J	**1.00***	0.13	0.11	0.49	0.24	**0.39***	
		K	0.06	0.10	0.09	0.19	0.15	0.12	
		L	0.15	0.07	0.08	0.09	0.07	0.09	
		M	0.06	0.15	0.19	0.11	0.13	0.13	
		N	0.08	0.09	0.15	0.24	0.32	0.18	
		O	0.07	0.04	0.06	0.10	0.07	0.07	
		Q	1.00	0.24	0.06	0.08	0.10	0.29	
	Data set 3	A	0.10	**0.30***	0.08	0.16	0.21	0.17	
		B	0.09	0.19	0.09	0.19	0.15	0.14	
		C	0.09	0.13	0.15	0.08	0.20	0.13	
		D	0.15	0.23	0.18	0.16	0.21	0.19	
		E	0.13	**0.30***	0.13	0.16	0.21	0.19	
		F	0.19	0.09	0.06	0.32	0.06	0.14	
		G	0.11	**0.29***	0.24	0.16	**0.54****	**0.27****	
		H	0.17	**0.31***	0.17	0.22	0.15	**0.20***	
		I	0.15	0.13	0.15	0.10	0.10	0.13	
		J	1.00	0.11	0.15	0.32	0.32	**0.38***	
		K	0.08	0.10	0.10	0.19	0.19	0.13	

Delimitation	Data set	OTU	BFI	GAPD	ILF3	MLC2A	RAG1	*egsi*	mtDNA
		L	0.10	0.04	0.08	0.11	0.09	0.08	
		M	0.06	0.24	0.07	0.15	0.49	0.20	
		N	0.06	0.09	0.19	0.32	0.32	0.20	
		O	0.08	0.10	0.32	0.06	0.24	0.16	
		Q	**1.00***	0.24	0.05	0.09	0.09	0.29	
	Data set 4	A	0.15	0.24	0.09	0.16	0.21	0.17	
		B	0.17	0.15	0.12	0.17	0.17	0.15	
		C	0.11	0.14	0.14	0.11	0.14	0.13	
		D	0.20	0.21	0.15	0.30	0.18	**0.21***	
		E	0.16	**0.28***	0.24	0.16	0.13	**0.19***	
		F	0.19	0.11	0.06	0.32	0.07	0.15	
		G	0.07	**1.00****	0.22	0.14	**0.65****	**0.42****	
		H	0.18	**0.34****	0.18	**0.27***	0.20	**0.23****	
		I	0.11	0.13	0.24	0.09	0.07	0.13	
		J	0.07	0.06	0.05	0.19	0.10	0.09	
		K	0.06	0.10	0.09	0.15	0.24	0.13	
		L	0.15	0.07	0.08	0.08	0.09	0.09	
		M	0.05	0.15	0.06	0.11	**1.00***	0.27	
		N	0.08	0.09	0.13	**1.00***	0.24	**0.31***	
		O	**1.00***	0.07	0.15	1.00	0.09	**0.46***	
		Q	**1.00***	0.32	0.08	0.09	0.11	**0.32***	
	Diploid	A	0.14	**0.25***	0.14	**0.25****	**0.27****	**0.21****	
		B	0.15	**0.24***	0.18	0.17	0.16	**0.18***	
		C	0.15	0.16	0.17	0.11	0.14	0.15	
		D	0.19	**0.31****	0.19	**0.25****	0.23	**0.24****	
		E	0.17	**0.34****	0.20	**0.25****	**0.28****	**0.25****	
		F	0.18	0.12	0.14	0.17	0.15	**0.15***	
		G	0.13	**0.44****	**0.26****	**0.22***	**0.73****	**0.36****	
		H	0.21	**0.45****	**0.24***	**0.25****	0.18	**0.26****	
		I	**0.28****	**0.28***	0.17	0.12	0.15	**0.20****	
		J	0.15	0.13	0.09	0.11	0.09	0.11	
		K	0.08	0.14	0.16	0.10	0.12	0.12	
		L	0.17	0.08	0.20	0.17	0.11	0.15	
		M	0.08	**0.21***	0.10	0.12	**0.26***	**0.16***	
		N	0.12	0.11	0.13	**0.42****	0.16	**0.19****	
		O	0.12	0.11	0.11	0.12	0.12	0.11	
		Q	**1.00****	0.10	0.09	0.10	0.10	**0.28****	
Geneland: 3 species	mtDNA + Data set 1	A	0.08	**0.50****	0.12	0.12	0.20	**0.20****	0.20
		B	0.12	**0.36****	0.26	**0.31***	0.22	**0.24****	0.17
		C	0.21	**0.40****	0.18	0.24	0.13	**0.22****	0.18
	mtDNA + Data set 2	A	0.16	**0.55****	0.20	0.20	**0.39****	**0.29****	0.20
		B	0.22	**0.38****	**0.38****	0.24	**0.66****	**0.34****	0.17
		C	0.20	**0.38****	**0.29***	**0.44****	**0.26****	**0.29****	0.18
	mtDNA + Data set 3	A	0.16	**0.50****	0.12	0.20	**0.39****	**0.26****	0.20
		B	0.19	**0.34****	**0.31***	**0.31***	**0.48****	**0.30****	0.17
		C	0.18	**0.27***	0.20	**0.29***	0.18	**0.22****	0.18
	mtDNA + Data set 4	A	0.08	**0.50****	0.16	0.08	**0.34****	**0.23****	0.20
		B	0.12	**0.28***	0.22	0.22	**0.28***	**0.21****	0.17
		C	0.20	**0.37****	0.22	**0.29***	0.17	**0.24****	0.18
	Diploid	A	0.14	**0.58****	0.16	0.18	**0.32****	**0.28****	
		B	0.19	**0.37****	**0.25***	**0.29****	**0.33****	**0.29****	
		C	0.23	**0.44****	**0.24***	**0.32****	**0.21***	**0.29****	

* $P < 0.01$

** $P < 0.001$
